# A step-channel gel polymer electrolyte for solid-state sodium batteries

**DOI:** 10.1093/nsr/nwag291

**Published:** 2026-05-26

**Authors:** Xiaoniu Guo, Shuai Guo, Shaohua Ge, Longfei Wen, Zhichao Gong, Yi Yang, Junyu Li, Ruixue Wang, Zhengkun Xie, Jiyu Zhang, Jun Luo, Jianqiang Kang, Lingfei Zhao, Jun Lu, Junhua Shao, Weihua Chen

**Affiliations:** College of Chemistry, Zhengzhou University, Zhengzhou 450001, China; College of Chemistry, Zhengzhou University, Zhengzhou 450001, China; College of Chemistry, Zhengzhou University, Zhengzhou 450001, China; College of Chemistry, Zhengzhou University, Zhengzhou 450001, China; Hubei Key Laboratory of Advanced Technology for Automotive Components, Wuhan University of Technology, Wuhan 430070, China; College of Chemistry, Zhengzhou University, Zhengzhou 450001, China; College of Chemistry, Zhengzhou University, Zhengzhou 450001, China; College of Chemistry, Zhengzhou University, Zhengzhou 450001, China; College of Chemistry, Zhengzhou University, Zhengzhou 450001, China; College of Chemistry, Zhengzhou University, Zhengzhou 450001, China; College of Chemistry, Zhengzhou University, Zhengzhou 450001, China; Hubei Key Laboratory of Advanced Technology for Automotive Components, Wuhan University of Technology, Wuhan 430070, China; Centre for Clean Energy Technology, School of Mathematical and Physical Sciences, University of Technology Sydney, Sydney NSW 2007, Australia; College of Chemical and Biological Engineering, Zhejiang University, Hangzhou 310027, China; Henan Finelyte New Energy Technology Co., Ltd., Jiaozuo 454000, China; College of Chemistry, Zhengzhou University, Zhengzhou 450001, China

**Keywords:** solid-state batteries, sodium batteries, step-channel gel polymer electrolytes, interface engineering, high energy density

## Abstract

Gel polymer electrolytes (GPEs) paired with abundant sodium (Na) and high-voltage polyanionic cathode, offer improved energy density and superior safety, positioning them as scalable alternatives for resource-limited lithium batteries. However, such technologies are plagued by critical interfacial engineering challenges: existing GPEs fail to sustain (electro)-chemical stability and mechanical close contact at high-loading cathodes and highly reactive anodes. Here, we report a rationally engineered GPE featuring biphasic polymer, creating step channels and polymer-solvent dipole adsorption to address the key issues. It enables volume-constrained bidirectional transport of infilling linear ether and cyclic carbonate solvents and endows the polymer with differential mechanical viscoelasticity for high-loading cathodes and ductile Na anodes. Therefore, a high-energy-density full cell of Na||Na_3_V_2_(PO_4_)_3_ (34.1 mg cm_cathode_^−2^, 194.4 Wh kg^−1^, based on total cell mass) and a stable pouch cell of 4.5 V-class Na||Na_2.466_Fe_1.724_Mg_0.043_(SO_4_)_3_ (18.7 mg cm_cathode_^−2^, ∼100% after 100 cycles) were demonstrated, with safety validation included. The design principles were established for this new chemical engineering pathway towards practical solid-state batteries.

## Teaser

This work provides a step-channel gel polymer electrolyte and its design principle to resolve interfacial (electro)-chemical and mechanical incompatibilities, enabling safe, stable, and high-energy-density solid-state sodium batteries for practical applications.

## INTRODUCTION

**Scheme 1.**
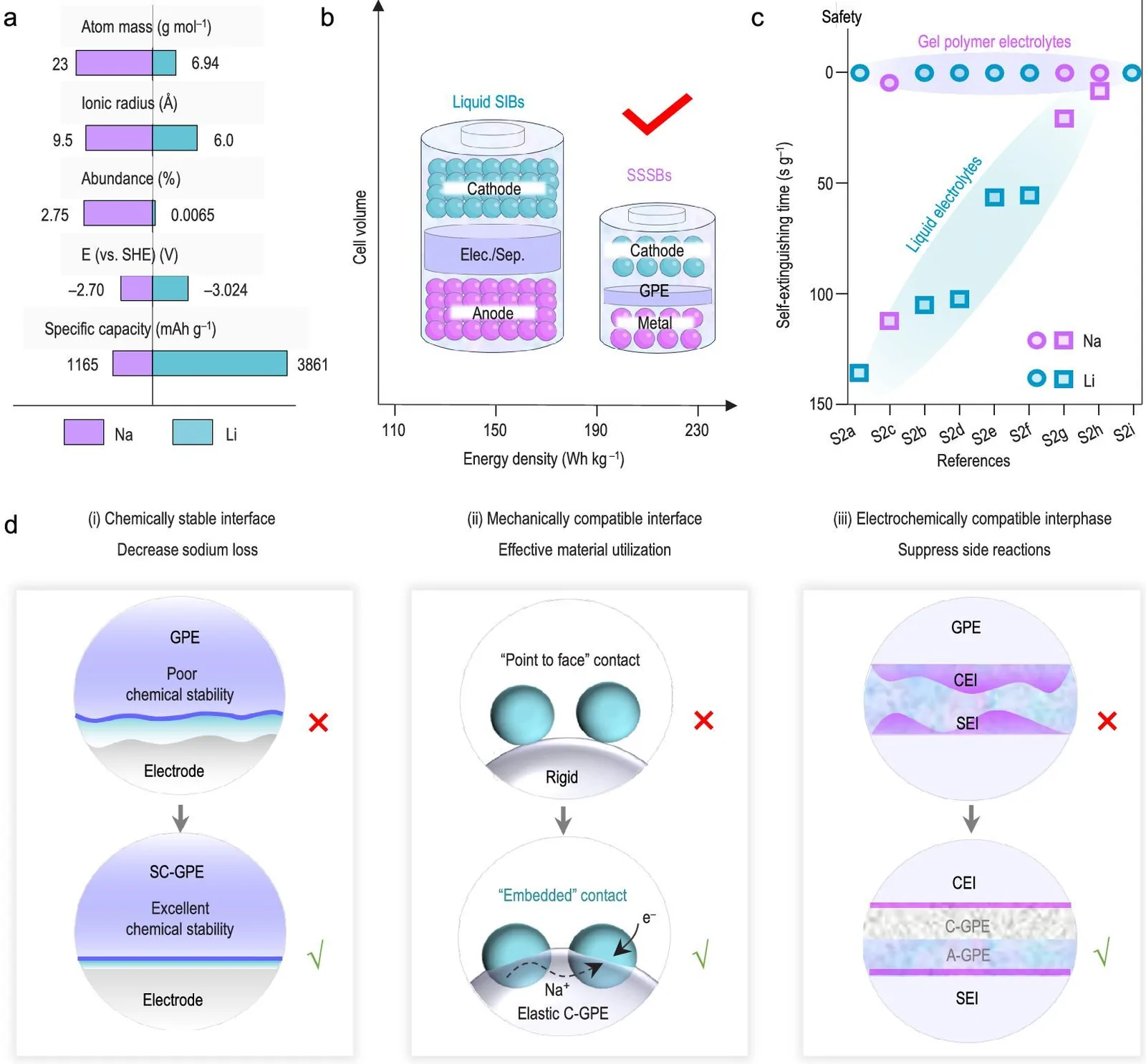
Importance of solid-state sodium batteries (SSSBs) and interface engineering requirements for GPE. (a) Comprehensive comparison of lithium and sodium properties. (b) Cell configurations for liquid sodium ion batteris (SIBs) and SSSBs. (c) Comparison of safety between liquid electrolytes and GPEs/SSEs in lithium/sodium batteries. (d) Key challenges in gel-state batteries: (i) (electro)-chemically unstable GPE, (ii) mechanical mismatch, and (iii) electrochemically incompatible interphase.

Practical solid-state sodium (Na) batteries (SSSBs) are attracting extensive interest for emerging artificial intelligence applications and large-scale sustainable energy storage, as they promise high energy density and superior safety, compared to resource-limited lithium-ion batteries (Table S1, Scheme 1a) [1–5]. Integrating solid-state electrolytes (SSEs) with high-capacity (1166 mAh g^−1^), low-potential (−2.71 V vs. standard hydrogen electrode (SHE)) Na anodes and high-voltage sodium iron sulfate cathodes can boost the overall energy density from 100–150 Wh kg^−1^ to about 200 Wh kg^−1^, significantly [6–9], narrowing the gap with commercial graphite||LiFePO_4_ systems (100–200 Wh kg^−1^; Scheme 1b and c). These advances have inspired great enthusiasm for the research on SSEs with high ionic conductivity and processability.

Gel polymer electrolytes (GPEs), outperforming all solid-state systems in conductivity and processability, are the preferred engineering pathway for developing SSEs [10,11]. However, the pursuit of high-safety and high-energy-density batteries in practical application demands that GPEs must overcome three key interfacial issues [12–14]: (i) Chemically unstable interfaces between GPEs and the reactive Na anode lead to irreversible Na loss. (ii) Mechanically mismatched interfaces between rigid GPEs and high-loading cathodes dramatically decrease active material utilization. (iii) Electrochemically fragile interfaces trigger GPEs oxidative/reductive decomposition at high-voltage cathodes (≥ 4.2 V vs. Na^+^/Na) and highly active Na anodes, sustaining continuous parasitic reactions (Scheme 1d). Various strategies have been proposed, including constructing dynamic bonding interactions to achieve interfacial self-healing [15], designing hetero-structured or MOF-incorporated GPEs to promote interfacial stability [16], and engineering polymer networks to regulate both bulk and interfacial properties [17]. But existing strategies often only optimize a single aspect of mechanical compatibility or electro-chemical stability [18–21]. A comprehensive understanding of engineered GPEs and rational design principles for electro-chemo-mechanical triple compatibility are still required to realize long-term applicability in next-generation SSSBs.

**Scheme 2.**
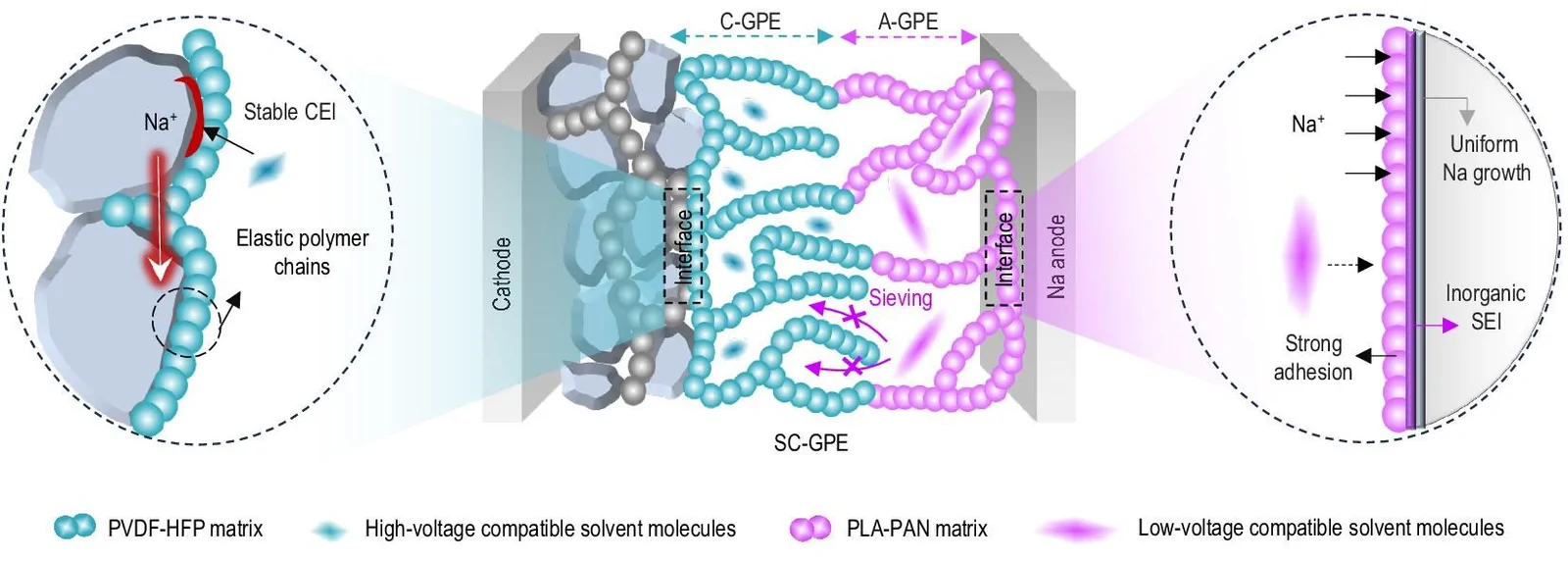
Schematic of step-channel GPE drives electro-chemo-mechanical adaptive interfaces for high-energy-density SSSBs.

Here we report a rationally engineered step-channel gel polymer electrolyte (SC-GPE) that concurrently resolves the interfacial electro-chemo-mechanical stability in SSSBs. The cathode-side poly(vinylidene fluoride-co-hexafluoropropylene (PVDF-HFP)-based matrix (C-GPE) can capture small-volume ester solvent molecules, creating a solvent-filled narrow channels. This sterically hinders the penetration of anode-targeted lone-pair electron ether solvent molecules, weakening their oxidation on the cathode-side. In contrast, the anode-side (A-GPE) employs a polylactic acid-polyacrylonitrile (PLA-PAN) scaffold with wide channels that admit bulky linear ether solvent molecules, reducing the likelihood of ester solvent molecules containing highly electronegative oxygen being reduced on the anode-side. The well-designed step-channel structure blocks deleterious crossover, thereby granting the SC-GPE outstanding interfacial chemical and electrochemical interfacial stability (Scheme 2). Through strong PVDF-HFP/ester compatibility for high-loading cathode-side elasticity and weak PLA-PAN/ether interactions for Na anode-side adhesion, this differentiated design ensures the mechanical compatibility of SC-GPE at both interfaces. As a result, the Na_3_V_2_(PO_4_)_3_ (NVP)-based solid-state Na-metal full cell demonstrates a practical energy density (34.1 mg cm_cathode_^−2^, 194.4 Wh kg^−1^, based on total cell mass). A solid-state Na-metal pouch cell of 4.5 V-class with Na_2.466_Fe_1.724_Mg_0.043_(SO_4_)_3_ (NFS)-based cathode retains 100% of the initial capacity after 100 cycles. Our work makes a critical step toward electrolytes through chemical engineering design for practical high-energy-density and safe SSSBs.

## RESULTS AND DISCUSSION

### Structure and properties of step-channel gel polymer electrolyte

**Figure 1.**
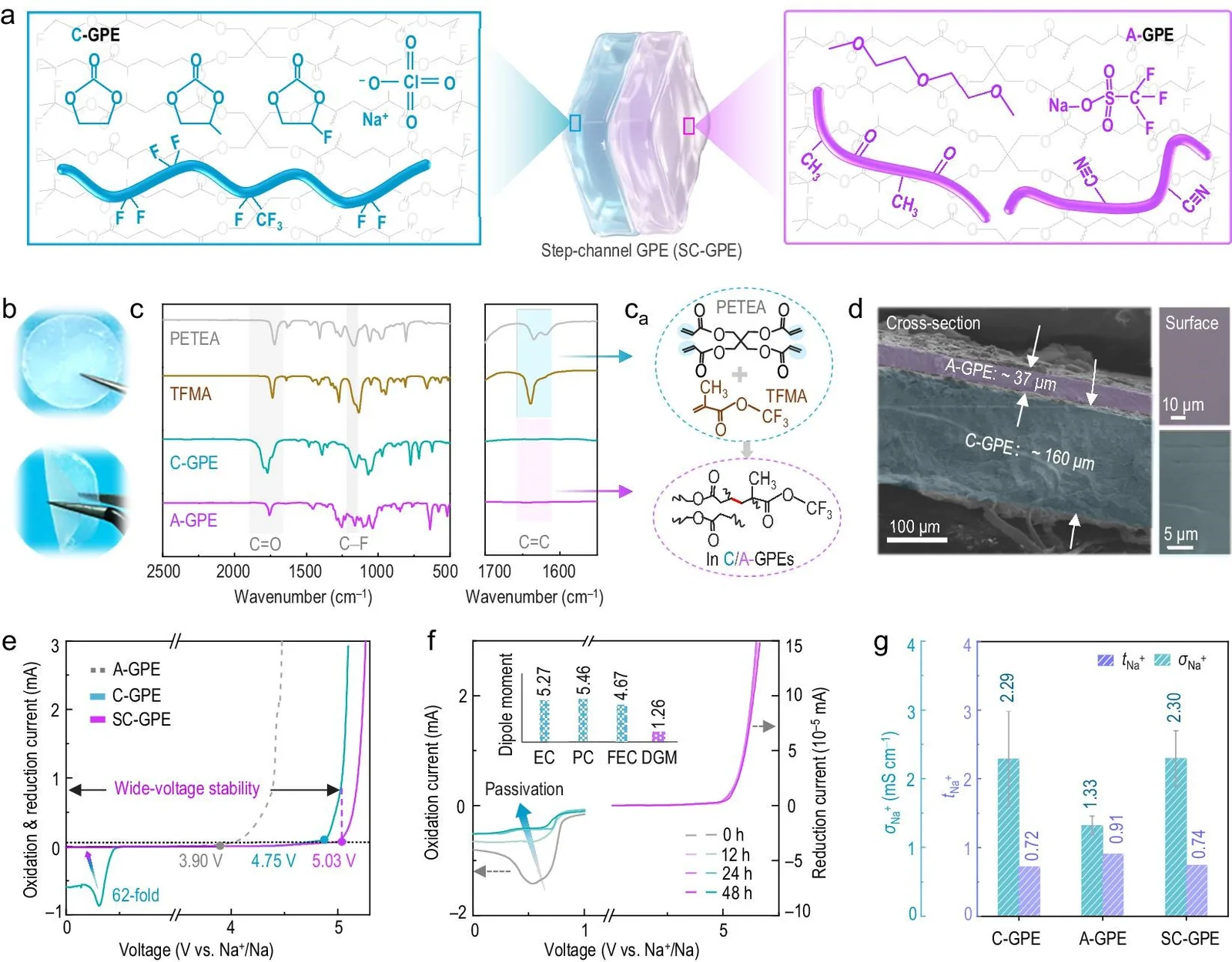
Structural design and physicochemical properties of SC-GPE. (a) Compositions of C-GPE and A-GPE in wide-voltage-range SC-GPE. (b) Optical images of flexible SC-GPE. (c) FTIR spectra of PETEA, TFMA monomers and C/A-GPEs, and (c_a_) monomer’s structures. (d) Cross-sectional and surface SEM images of the SC-GPE. (e) LSV of C/A-GPEs and SC-GPE in SS||Na cells and (f) long-term storage stability. (g) The Na^+^ transference number and Na^+^ conductivity of C-GPE, A-GPE and SC-GPE.

The SC-GPE integrates a PVDF-HFP polymer matrix with weak polarity and a PLA-PAN polymer matrix with strong polarity. Specifically, the SC-GPE was fabricated through UV polymerization, among which, the C-GPE—containing trifluoroethyl methacrylate (TFMA), pentaerythritol tetraacrylate (PETEA), and ester-based molecules (ethylene carbonate (EC), propylene carbonate (PC), diethyl carbonate (DEC), fluoroethylene carbonate (FEC))—was polymerized in PVDF-HFP matrix (Fig. 1a). The PLA-PAN layer was then plasticized by A-GPE precursor (TFMA, PETEA, and ether-based molecules (diethylene glycol dimethyl ether (DGM), tetraethylene glycol dimethyl ether (TEGDME)) and polymerized atop C-GPE (Fig. 1b). For further studies, 1 M NaClO_4_ in EC/PC with 5 wt% FEC and 1 M NaCF_3_SO_3_ in DGM were chosen as the representative electrolytes in precursor. Fourier-transform infrared spectroscopy (FTIR) analysis confirmed gelation, shown by the disappearance of unsaturated C=C bonds in TFMA (1638.6 cm^−1^) and PETEA (1630.9 cm^−1^) after polymerization (Fig. 1c and c_a_, and Figs S1–S3). Cross-sectional scanning electron microscopy (SEM) images (Fig. 1d) revealed dense surface morphologies for C-/A-GPEs (Fig. S4), with thickness of 160 μm and 37 μm, respectively. The thicker C-GPE was designed to increase the diffusion distance of DGM, thereby inhibiting its migration toward the cathode.

The Na^+^ transport kinetics and interface stability of SC-GPE were evaluated through experiment and molecular dynamics (MD) simulation. The A-GPE exhibited oxidative instability above 3.90 V (vs. Na^+^/Na) due to the susceptibility of oxygen's lone pairs, while C-GPE underwent severely reductive decomposition below 0.90 V, resulting in narrow electrochemical stability windows of 3.90 and 4.00 V (Fig. 1e, values derived from the voltage at 0.05 mA), respectively. This bidirectional instability renders single-component GPEs unsuitable for the asymmetric voltage requirements in SSSBs [22]. In contrast, the SC-GPE integrated C-GPE’s high-voltage stability and A-GPE’s Na anode compatibility. The SC-GPE suppressed C-GPE’s reduction current by 62-fold while maintaining excellent oxidative stability (Figs S5 and S6). Time-dependent linear sweep voltammetry (LSV) measurements (after 0–48 h of storage, Fig. 1f) showed no detectable DGM decomposition. Even with increasing parasitic reactions between high-polarity EC/PC/FEC molecules and Na metal over time, the SC-GPE still maintained enhanced chemical and electrochemical stability compared to C-GPE alone. X-ray photoelectron spectroscopy (XPS) analysis detected no S-containing species (from NaCF_3_SO_3_) on the NFS side and only trace amount of Cl (from NaClO_4_) on the Na anode side (Fig. S7), consistent with LSV results. The Na^+^ transference number (*t*_Na⁺_; Fig. S8) was 0.72 for C-GPE and 0.74 for SC-GPE, both lower than that of A-GPE (0.91) (Fig. 1g). The SC-GPE achieved Na^+^ conductivity of 2.30 mS cm^−1^, comparable to that of C-GPE (2.29 mS cm^−1^) and higher than that of A-GPE (1.33 mS cm^−1^). Thus, SC-GPE can effectively restrict solvent molecule transport while maintaining high Na^+^ conductivity.

### Transport mechanisms of solvent molecules and Na ions in SC-GPE

Within a GPE, polymer matrix serves as the robust framework and solvent molecule diffusion occurs exclusively within liquid-phase microdomains, which are essential channels (free volume) formed between polymer chains for facilitating Na^+^/solvent transport and accommodating chain dynamics. Na^+^ transport relies on electromigration through polymer segments and liquid-phase microdomains. The step-channel structure enables selective solvent screening, analogous to the ‘sorption-diffusion-desorption’ process in gas separation membranes [23], governed by:


(1) \begin{eqnarray*}
P = D \times S,
\end{eqnarray*}

(2) \begin{eqnarray*}
D \propto \exp \left( {\frac{{ - V_{{\rm molecule}}^ * }}{{{f}_{{\rm polymer}}}}} \right),
\end{eqnarray*}

where *P* represents the permeability, *D* represents the diffusion coefficient, *S* represents the solubility coefficient (reflecting functional group-solvent affinity) (Equations 1 and 2), *f*_polymer_ represents the free volume of GPE, while *V*^∗^_molecule_ represents the volume of solvent molecules. The formation of gels benefits from the spontaneous thermodynamic nature of interactions between the solvent molecules and polymers (Fig. S9), which diminishes the relative influence of *S*. As a result, the comparison between *V*^∗^_molecule_ and *f*_polymer_ is the key factor for influencing *P*, i.e. the interfacial electrochemical stability of SC-GPE.

**Figure 2.**
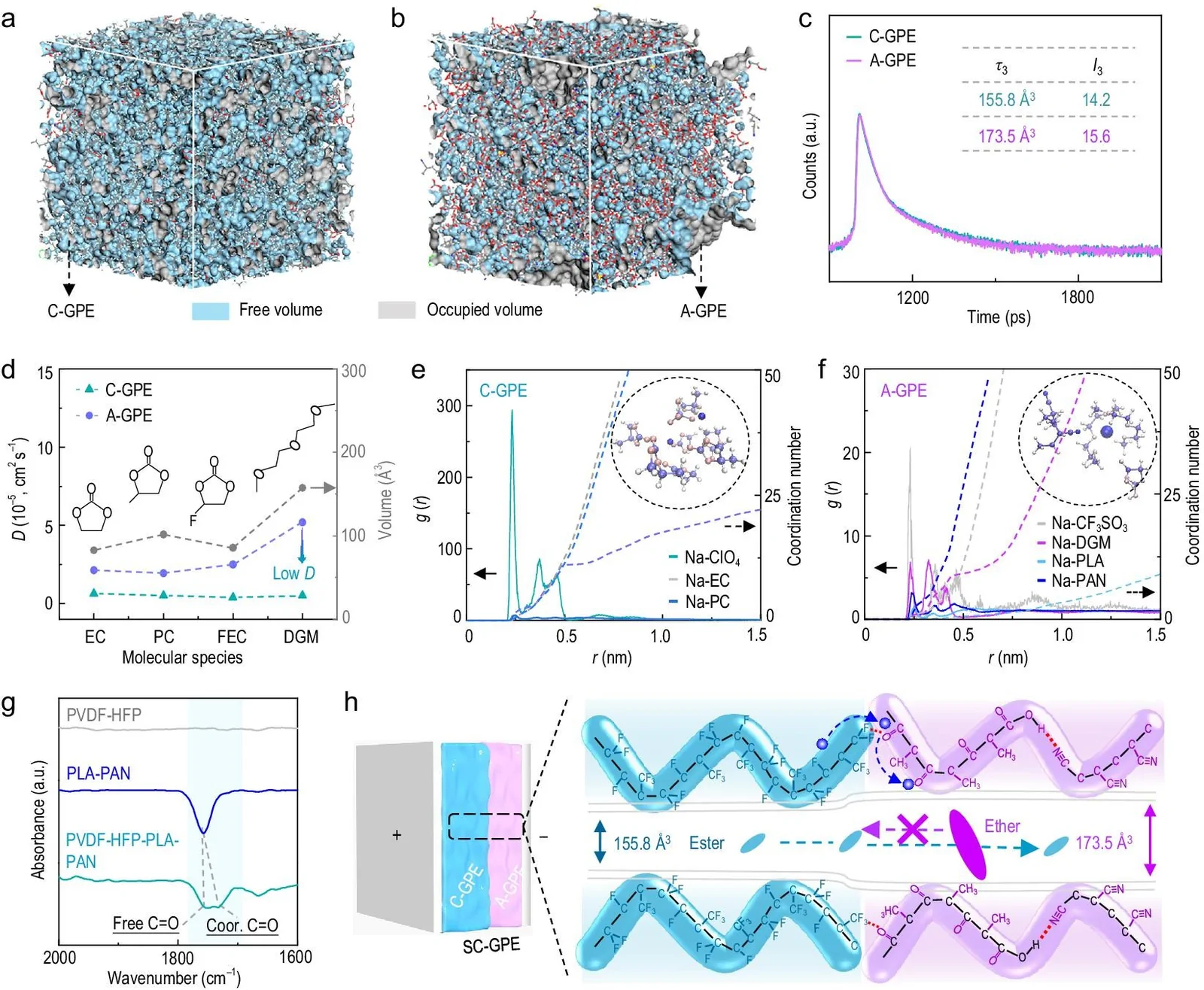
Polymer-regulated solvent sieving and Na^+^ diffusion mechanism in SC-GPE. (a, b) Free-volume characteristics analyzed by MD simulation and (c) positron annihilation spectroscopy. (d) Solvent diffusion properties and molecular volume effects. Radial distribution function and the coordination number for (e) C-GPE and (f) A-GPE. (g) FTIR spectra of polymer matrices in C-GPE and A-GPE. (h) Schematic diagram of the interaction between polymer matrices and their solvent-sieving functionality.

The positron annihilation lifetime spectroscopy (PALS) and MD simulation reveal a size-dependent transport behavior [24], which is governed by the polymer’s channel structure. MD simulations visualize these channels (blue-green areas) in C-GPE and A-GPE (Fig. 2a and b). In contrast, quantitatively PALS analysis indicates that C-GPE possesses a smaller accessible free volume (155.8 Å^3^) compared to A-GPE (173.5 Å^3^) (Fig. 2c). Consequently, C-GPE effectively blocks bulky DGM (157.6 Å^3^, *V*_DGM_ > *f*_C-GPE_), resulting in a sharp decline of permeation (*P*). On the other hand, the larger accessible channels in A-GPE (173.5 Å^3^) accommodate smaller cyclic ester solvents (EC: 82.7, PC: 101.7, FEC: 82.5 Å^3^; Fig. 2d and Fig. S10), although their permeation and reductive activity are suppressed (62 times lower) due to resistance from the polymer matrix and its functional groups. MD simulations confirm DGM’s diffusion coefficient in C-GPE (5.20 × 10^−6^ cm^2^ s^−1^) is one order of magnitude lower than that in A-GPE (5.02 × 10^−5^ cm^2^ s^−1^) (Fig. S11), definitely linking narrow channels to obstructed solvent diffusion.

Although linear DGM is theoretically expected to exhibit higher Na^+^ transport kinetics than cyclic EC/PC/FEC electrolytes due to its low viscosity and weak coordination [25,26], polymer chains alter this behavior [24]. In C-GPE, C–F bonds induce weak Na^+^ coordination and are excluded from the solvation structure, forming ([Na^+^][ClO_4_^−^][EC]_3.84_[PC]_3.65_) clusters. Conversely, electron-rich –C=O and –C≡N groups of PLA-PAN in A-GPE strongly coordinate Na^+^, resulting in polymer-involved solvation clusters ([Na^+^][CF_3_SO_3_^−^][DGM]_1.95_[PAN]_1.10_[PLA]_3.13_; Fig. 2e and f), which leads to a lower Na^+^ conductivity of A-GPE (1.33 mS cm⁻^1^) than that of C-GPE (2.29 mS cm^−1^). Thus, the A-GPE layer fails to boost the Na^+^-transport kinetics of SC-GPE (Figs S12 and S13), which are predominantly governed by the C-GPE main layer. These findings provide a molecular-level explanation for the differences in ion transport kinetics of GPEs. Smooth Na^+^ conduction between C-GPE and A-GPE interface is ensured by bonding bridges and shared monomer filling. Compared to PLA-PAN, the split C=O peak in PVDF-HFP–PLA-PAN composite (Fig. 2g) indicates an interaction between polar C–F and C=O groups [27]. The synergy of interfacial engineering and step-channel design enables efficient Na⁺ conduction, selective solvent sieving, and collectively enhanced interfacial compatibility (Fig. 2h).

### Mechanical adaptability of SC-GPE

The interfacial contact integrity of GPE is critical for the stability of SSSBs [28,29]. The leakage of liquid solvent can maintain initial solid-solid interfacial contact, yet it significantly compromises safety and cannot support long-term operation due to continuous electrolyte depletion. In contrast, the adaptive capability of polymer chains to high-loading cathodes determines durable interfacial contact and stable battery cycling [30]. Three key parameters govern this: 1) Stress-strain response, reflecting the macroscopic deformation behavior and pressure tolerance under external forces. 2) Viscoelasticity refers to the viscous dissipation (from solvent-plasticized interchain friction) and elastic recovery (via segment rearrangement and stress release), enabling adaption to electrode volume changes (measurable by dynamic mechanical thermal analysis, DMTA). 3) Flexibility, guided by free volume theory, where loosely structured polymer architectures facilitate chain rotation and improve electrode contact.

**Figure 3.**
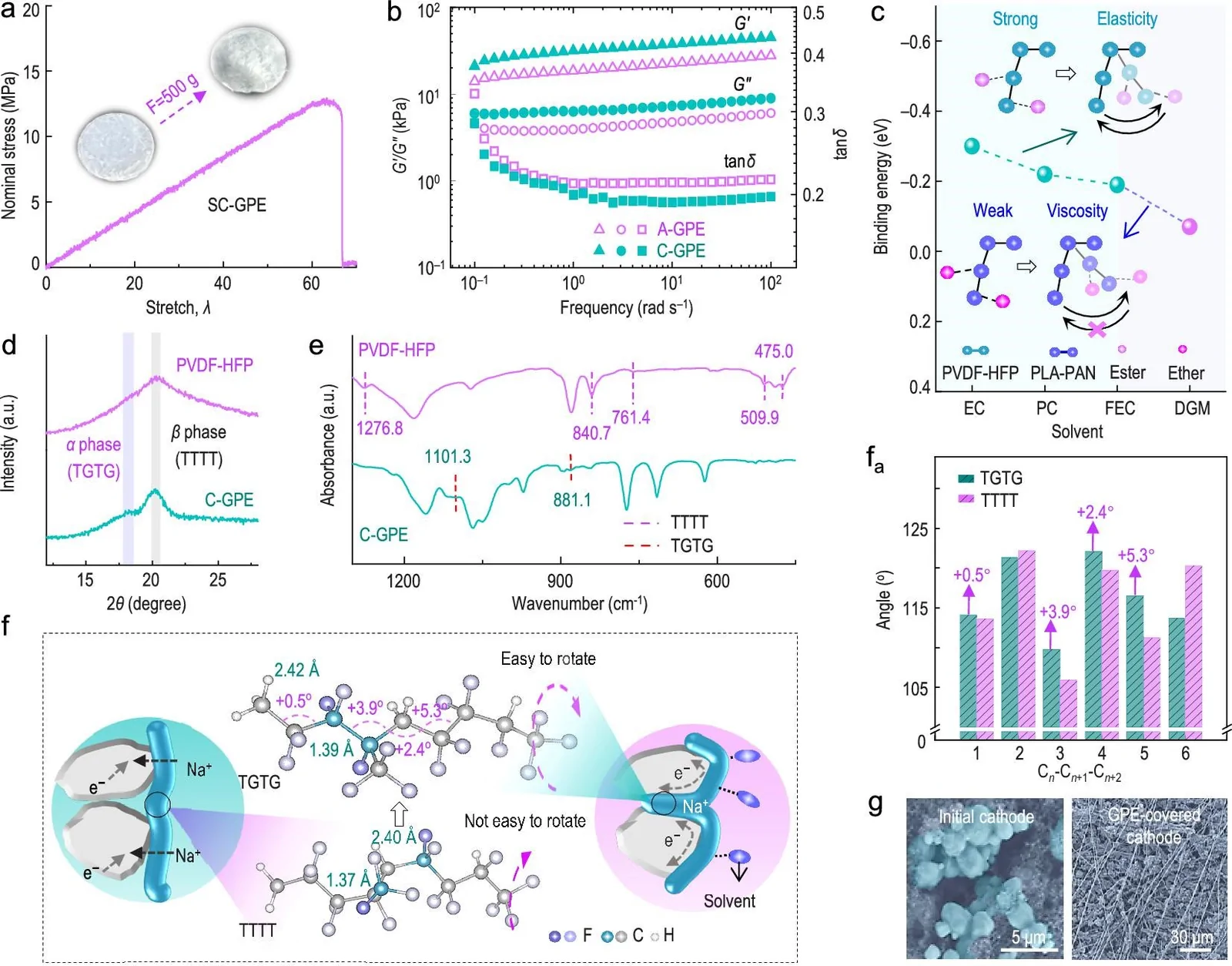
Mechanical adaptability of SC-GPE tailored by polymer-solvent interactions. (a) Strain-stress curve of SC-GPE. The inset shows mechanical toughness test of SC-GPE. (b) Dynamic mechanical analysis profiles of C-/A-GPEs. (c) Binding energies between solvents and PVDF-HFP/PLA-PAN polymers. The inset illustrates the regulation of chain motion by interactions. (d) X-ray diffraction (XRD) patterns of PVDF-HFP and C-GPE. (e) FTIR spectra of PVDF-HFP and C-GPE. (f) Schematic of rigid and flexible polymer chains in contact with electrode. (f_a_) Bond angle changes in TGTG and TTTT conformations. (g) SEM images of initial and SC-GPE-covered cathode.

A systematic analysis was conducted to evaluate how the polymer molecular structure influences macro-to-micro mechanical properties of SC-GPE. Stress-strain analysis (Fig. 3a) demonstrates that SC-GPE exhibits remarkable mechanical toughness. It initially undergoes elastic deformation, following Hooke’s Law, and shows a modulus of 21.44 MPa. Beyond the elastic limit, the SC-GPE displays high tensile strength (12.92 MPa) and toughness (459.2 kJ m^−3^). Notably, the SC-GPE maintains structural integrity under a 500 g load (Fig. 3a, inset) without interlayer cracking, demonstrating its excellent processability. Rheological analysis revealed distinct viscoelastic behaviors between the C-GPE and A-GPE (Fig. 3b). Both layers exhibit stable storage modulus (*G′*) and loss modulus (*G″*), with a loss tangent (tan*δ*) < 1, confirming the elastic solid-like characteristics. The consistently higher *G″* than *G′* indicates efficient stress dissipation and strong adaptability to electrode deformation [31]. Notably, the A-GPE demonstrates a greater tan*δ* than C-GPE, reflecting its relatively viscous behavior. In contrast, the lower tan*δ* suggests enhanced elasticity, which facilitates stress release. These difference stems from distinct polymer-solvent interactions. Strong PVDF-HFP-EC/PC/FEC interactions promote stable cross-linked networks, enabling elastic recovery upon deformation, whereas weak PLA-PAN-DGM interactions lead to discontinuous networks, where energy dissipates via irreversible polymer chains-solvents friction, resulting in the viscosity (Fig. 3c) [32].

Polymer flexibility, derived from the chain segment's rotational freedom, enhances electrode-GPE interfacial contact, as validated via bond angle and length analysis. In C-GPE, PVDF-HFP undergoes a conformational transition, evidenced by the emergence of a new *β*-phase (TTTT conformation) diffraction peak at 18.4° alongside a residual *α*-phase (TGTG conformation) signal at 20.4° (Fig. 3d, XRD); this transformation was also accompanied by the appearance of TGTG peaks at 881.1 cm^−1^ and 1101.3 cm^−1^ (Fig. 3e, FTIR spectroscopy) [33]. Molecular modeling revealed distinct bond angles and lengths for each conformation [34]. The TGTG conformation shows elongated C–F bonds (1.39 Å, 2.42 Å, compared to 1.37 Å and 2.40 Å in TTTT) and widened bond angles (e.g. C_3_-C_4_-C_5_ : 109.8° vs. 105.9°; C_5_-C_6_-C_7_ : 116.5° vs. 111.2°). These structural adjustments reflected an extended, low-energy chain architecture that facilitated segment rotation, resulting in increased flexibility and improved cathode adhesion for long-term cycling stability (Fig. 3f and f_a_). The SC-GPE achieves multidimensional mechanical properties that are critical for stable battery cycling. This optimization enables homogeneous deformation under stress without interfacial delamination, confirming strong interlayer cohesion. Post-cycling analysis confirmed these advantages, showing that NFS cathode particles remain fully encapsulated within the SC-GPE matrix (Fig. 3g). The synergistic integration of high strength, tailored viscoelasticity, and structural flexibility collectively ensures the long-term reliability of SSSBs.

### Interfacial chemistry of SC-GPE

**Figure 4.**
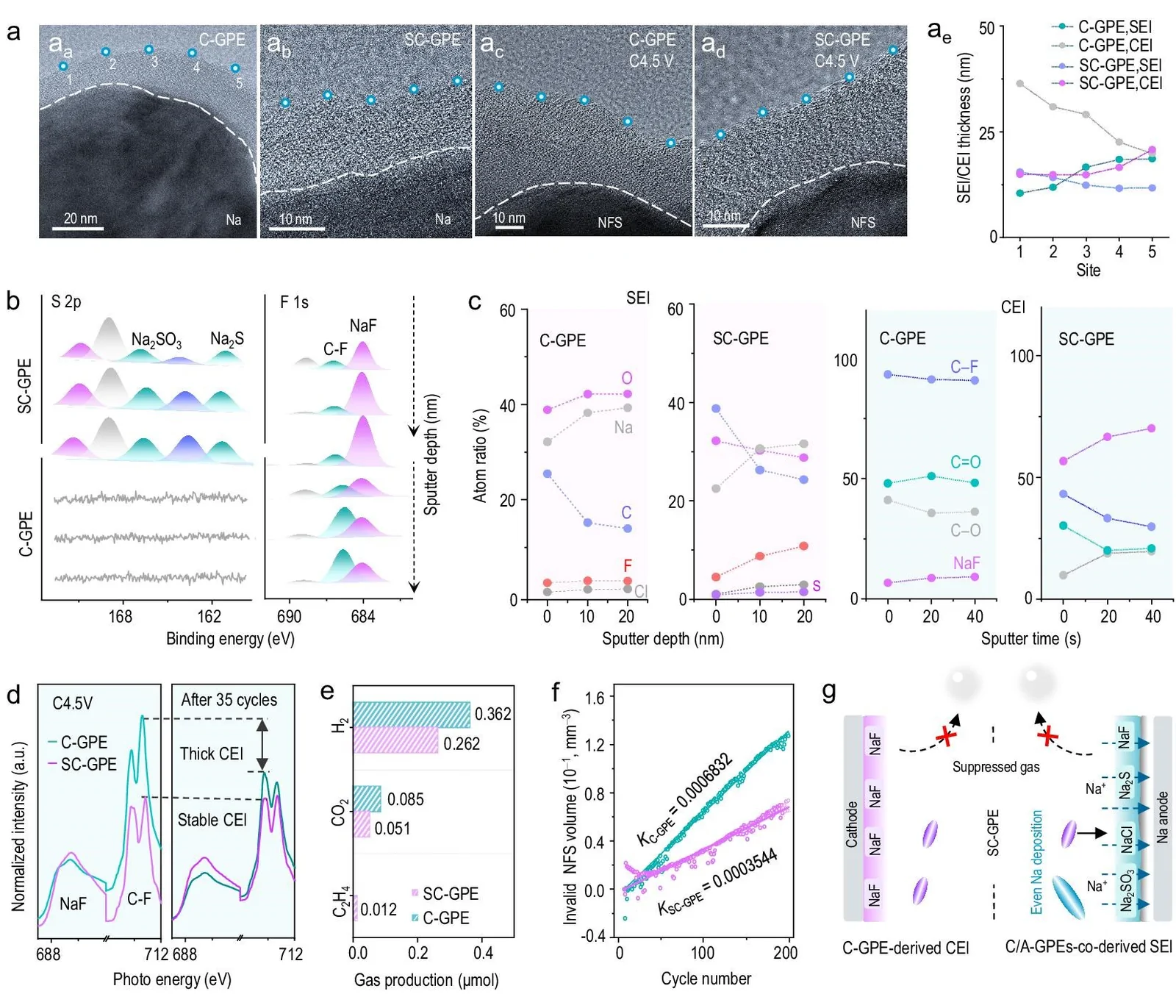
Interface chemistry with SC-GPE. (a) Cryo-TEM images of SEI (a_a_–a_b_) and CEI (a_c_–a_d_) with corresponding thickness distribution (a_e_). (b) Depth-profiling XPS spectra (S 2p, F 1s) of cycled Na anodes at different sputtering times (0 s, 30 s, 60 s). (c) Comparative XPS analysis showing (left) elemental composition of SEI on Na anode and (right) depth distribution of C–F/NaF and C–O/C=O components on NFS cathode. (d) F K-edge and C K-edge XAS spectra for NFS cathode at 4.5 V and after 35 cycles. (e) Comparative analysis of gas evolution in Na||NFS cells during initial 10 cycles. (f) Quantitative assessment of inactive NFS volume variation during cycling. (g) Schematic illustration of stabilized SEI/CEI formation enabled by SC-GPE.

The solvent-restricted SC-GPE facilitates precise interphase regulation, prompting a systematic investigation of CEI/SEI evolution. Cryogenic transmission electron microscopy (Cryo-TEM) analysis reveals that SC-GPE forms a uniform SEI (11.6–15.5 nm) on Na anode surface, which is significantly thinner than C-GPE-derived SEI (10.5–18.6 nm; Fig. 4a_a_, a_b_, and a_e_). Depth-profiling XPS spectra (S 2p, F 1s) elucidate the SEI’s compositional evolution (Fig. 4b). In SC-GPE system, the F 1s spectra demonstrate a gradual increase in F content, ultimately dominating the F 1s spectra, confirming an inorganic-F-rich SEI structure. In contrast, C-GPE exhibits C–F bond dominance. Notably, significant S species derived from NaCF_3_SO_3_ is observed. The presence of Na_2_S and Na_2_SO_3_ indicates DGM participation in SEI formation. SC-GPE-derived SEI has trace Cl ratio (∼1.09%, minor ester diffusion) and features homogeneous, inorganic-rich, and high-entropy characteristics (Fig. 4c), which are conducive to Na deposition/stripping kinetics and suppress dendritic Na [35,36]. Under 51 minutes of Na deposition at 1.0 mA cm^−2^, the viscous A-GPE in SC-GPE and inorganic-rich SEI synergistically enable uniform Na deposition (Fig. S14).

The superior interface stability of the designed SC-GPE was further confirmed through comprehensive CEI characterizations. Cryo-TEM analysis shows that SC-GPE induces thin and uniform CEI (14.8–20.8 nm), in stark contrast to the defective and thicker CEI (19.8–36.4 nm) generated by C-GPE, which triggers more parasitic side reactions (Fig. 4a_e_). Post-cycling analysis demonstrates the CEI stability in Na||NFS cells with SC-GPE, maintaining a CEI thickness of 15.6–18.5 nm, whereas C-GPE exhibits significant thickening, 25.9–29.1 nm after 35 cycles (Fig. 4a_c_ and a_d_, and Fig. S15). Depth-profiled XPS reveals compositional differences. The SC-GPE system shows a substantial increase in NaF content with etching depth (from 56.74% to 70.17%), while C-GPE maintains consistently lower NaF contents (from 6.71% to 9.17%). This inorganic-rich nature is further evidenced by lower C content (C–O/C=O: 19.70%/20.97% for SC-GPE vs. 36.27%/48.25% for C-GPE) (Fig. 4c). Driven by partial solvent migration to Na anode, the inorganic-rich CEI forms preferentially due to promoted decomposition of solvation clusters at NFS cathode. X-ray absorption spectroscopy (XAS) further reveals stronger NaF signals (690.9 eV) and weaker C–F features (707.5/709.2 eV) (Fig. 4d). After 35 cycles, the attenuation of NaF and C–F peak intensities confirms CEI thickening, which aligns well with the Cryo-TEM observations.

In-situ differential electrochemical mass spectrometry (DEMS) results confirm the effective suppression of gas evolution by the SC-GPE, which exhibits significantly reduced H_2_ (0.362 vs. 0.262 μmol) and CO_2_ (0.085 vs. 0.051 μmol) during the initial 10 cycles compared to C-GPE (Fig. 4e, Fig. S16a and b). The C_2_H_4_ (0.012 μmol)-derived from DGM further indicates the A-GPE-mediated the formation of SEI (Fig. S17 (Ⅰ–Ⅱ)) [37–39]. Additionally, C-GPE-assembled cells display pulsed gas peaks, reflecting incomplete interfaces, whereas SC-GPE facilitates complete electrode-SC-GPE interfaces that shifts the accelerated H_2_ evolution threshold from 3.55 V to 3.87 V (Fig. S16c and d). The formation of gas bubbles could otherwise lead to electrochemically inactive ‘dead zones’, which degrade battery cycling. Quantitative analysis based on the pseudo two-dimensional model (P2D, Fig. 4f) shows a slower growth rate (*K* = 0.0003544) of invalid NFS volume per cycle relative to the C-GPE (*K* = 0.0006832), along with higher effective porosity (Fig. S18a and b), which collectively help preserve more electrochemically active sites. These results strongly demonstrate that SC-GPE formed the electro-chemo-mechanical optimized interfaces (Fig. 4g).

### Electrochemical performance of solid-state sodium batteries with SC-GPE

**Figure 5.**
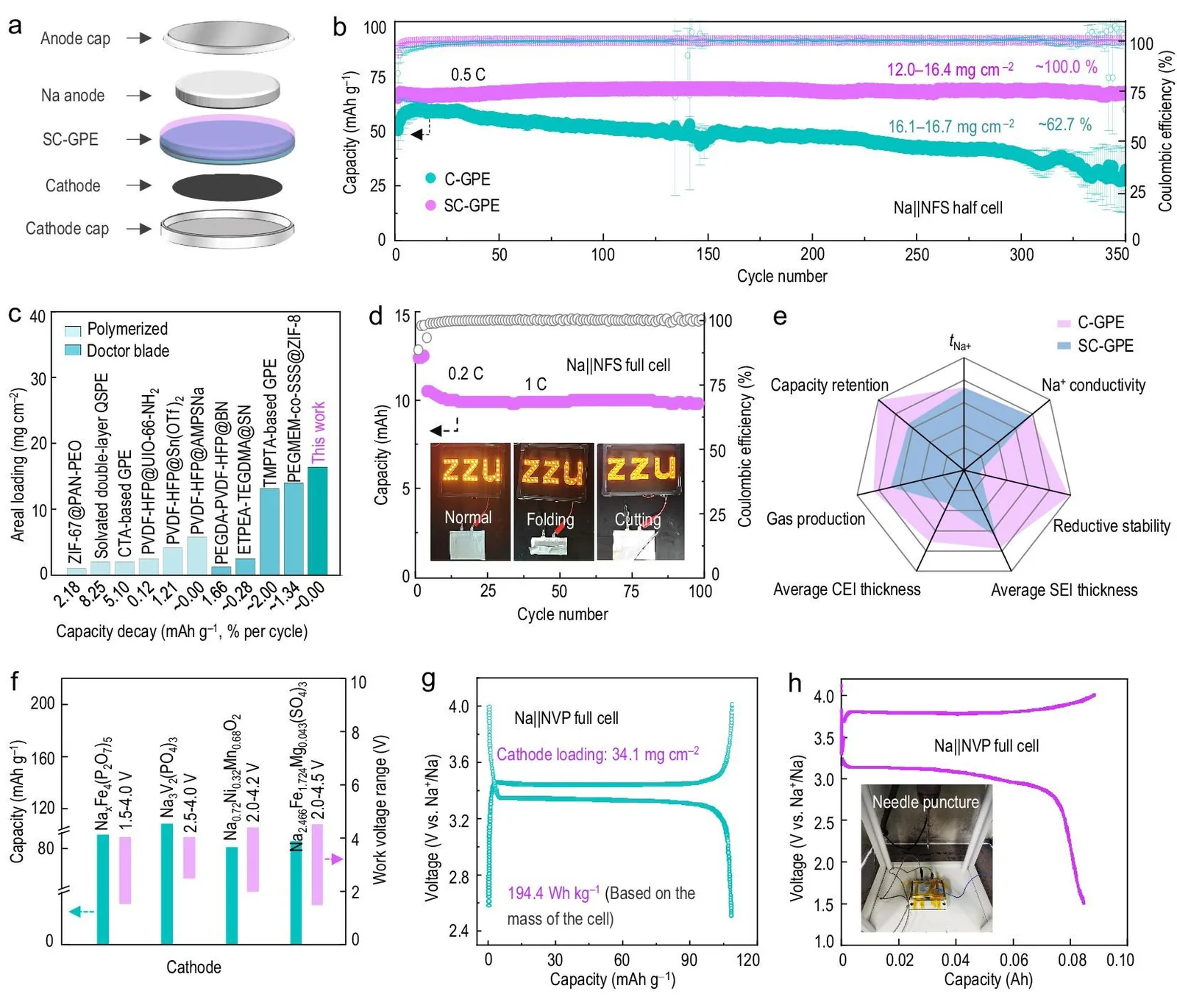
Electrochemical and safety performances of SC-GPE. (a) Schematic diagram of battery configuration. (b) Cycling performance of Na||NFS half cells. The error bars come from four cells. (c) Comparison of capacity decay with most previously reported works. (d) Cycling performance of the Na||NFS pouch cell with SC-GPE. Inset: the photograph of the pouch cell under different conditions. (e) Comparison of comprehensive performance between C-GPE and SC-GPE. (f) Cathode compatibility of SC-GPE and their work voltage. (g) Capacity-voltage plots of the Na||NVP full cell and (h) pouch cell using SC-GPE. Inset: the photograph of a pouch cell under nail puncture conditions.

The electrochemical compatibility of the SC-GPE was evaluated in a Na||NFS system (2.0–4.5 V) (Fig. 5a), which uses a cost-effective, sustainable NFS cathode based on earth-abundant elements, aligning well with the principles of sustainable energy storage. The Na||NFS half cell with SC-GPE exhibits 93.0% capacity retention after 200 cycles at 1 C (Fig. S19), significantly better than C-GPE (85.8% retention), aligning with the cyclic voltammetry in Fig. S20. Furthermore, the Na||NFS half cell incorporating the SC-GPE delivers comparable capacities across a range of mass loadings (2.27–8.67 mg cm^−2^; Fig. S21) all while maintaining stable cycling. Even within the high areal loading range of 12.0 mg cm^−2^ to 16.4 mg cm^−2^, the cell retains nearly 100% of its capacity after 350 cycles at 0.5 C (Fig. 5b and Fig. S22a), surpassing most reported GPEs in sodium batteries (Fig. 5c and Table S2). SEM images show that the A-GPE and C-GPE layers remain tightly bonded after 35 cycles, supporting stable battery cycling (Fig. S23). The polarization voltage decreases gradually from 1.02 V in the first cycle to approximately 0.87 V, stabilizing after 50 cycles, suggesting ongoing interface reorganization and improved electrode-SC-GPE contact (Fig. S22b–d). In contrast, C-GPE assembled half cell (cathode areal loading: 16.1–16.7 mg cm^−2^) experience gradual capacity decay (62.7% retention). Rate capability tests show that the C-GPE half cell delivered discharge capacities of 69.3, 63.8, 55.1, 46.7, and 41.0 mAh g^−1^, significantly lower than the 76.1, 70.5, 64.9, 51.4, and 46.1 mAh g⁻^1^ achieved with the SC-GPE (Fig. S24). The superior rate performance of the SC-GPE is closely linked to its stable and uniform ion transport pathways. The Na||Na cell with SC-GPE exhibits a decreased polarization voltage (from 29 to 17 mV at 0.2 mA cm^−2^) due to electrode activation, stably cycling for 1000 h (Fig. S25a). In contrast, the C-GPE shows voltage fluctuations between 300 and 400 h, indicating SEI instability, and suffers a dendrite-induced short circuit after 627 h. The SC-GPE achieves a high critical current density of 3.1 mA cm^−2^ (Fig. S25b), enhancing the practicality for Na metal batteries.

The designed SC-GPE assembled Na||NFS pouch cell delivers an energy density of 191.3 Wh kg^−1^ (based on the mass of NFS and Na anode) at 0.2 C with an N/P ratio of 2.0 (Fig. 5d and Table S3). Overall, the comprehensive performance of SC-GPE exceeds C-GPE (Fig. 5e). Concurrently, the Na||NFS pouch cell exhibits impressive cycling stability, retaining 93.4% of its capacity over 100 cycles. Meanwhile, its electrochemical performance at 0°C and 60°C was further tested (Fig. S26a–d). The SC-GPE also shows broad applicability by supporting stable cycling in other cathode systems such as Na*_x_*Fe_4_(P_2_O_7_)_5_ (1.5–4.0 V; Fig. S27), Na_0.72_Ni_0.32_Mn_0.68_O_2_ (2.5–4.0 V; Fig. S28). It can also be compatible with the widely used commercial NVP cathode (2.5–4.0 V; Fig. 5f and Fig. S29a–c) between –10 and 60°C. The assembled Na||NVP coin full cell with ultra-high NVP loading (34.1 mg cm^−2^; Fig. S30 and Table S4) maintains 99.02% capacity retention over 40 cycles (Fig. 5g and Fig. S31), delivering a high energy density of 194.4 Wh kg^−1^ based on total cell mass excluding casing and 345.6 Wh kg^−1^ based on the total mass of active materials (Table 1), Na anode weight excludes Al current collector, comprising only Na metal. Importantly, the Na||NVP pouch cell exhibits 152.08 Wh kg^−1^ energy density based on total cell mass excluding casing and 271.59 Wh kg^−1^ based on the electrode mass (Fig. 5h and Table 1), exhibiting high energy density. Safety tests further confirm the practical value of the SC-GPE. In a fully charged Na||NVP pouch cells, a 10-minute short circuit caused only a 0.7°C temperature rise over 1 hour (Fig. S32a–d). The cell also passed a nail penetration test, maintaining a maximum temperature of 26.9°C with minimal voltage fluctuation (Fig. S32e–f, the inset of Fig. 5h), further confirming its practical safety.

**Table 1.**
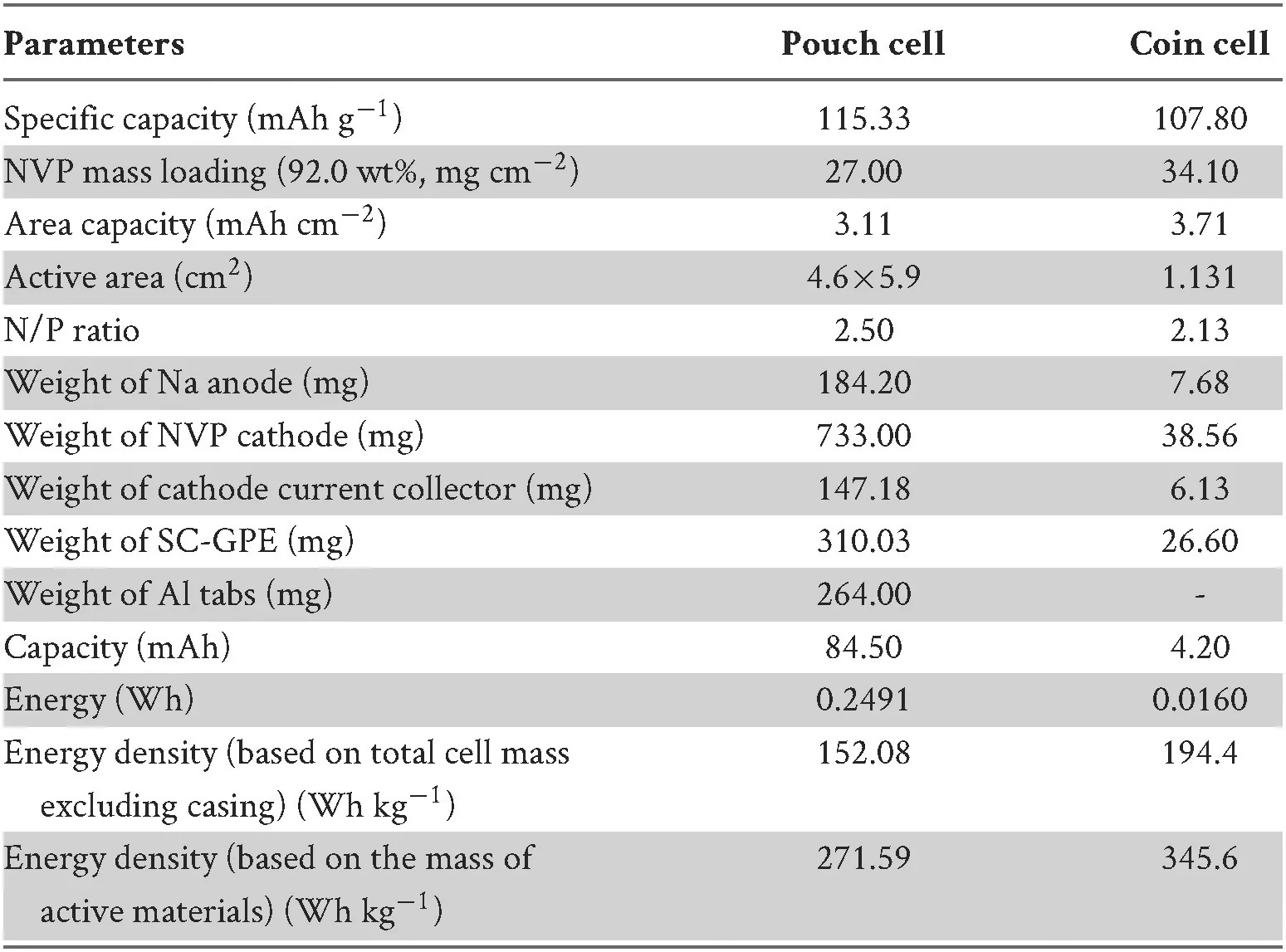
Parameters of Na||NVP full cells.

### Design principles of SC-GPE for solid-state batteries

A systematic investigation into SC-GPE’s design principles is conducted. According to Equations 1 and 2, the diffusion coefficient *D* exhibits an exponential relationship with the free volume of polymer (*f*_polymer_) and solvent volume (*V*^∗^_molecule_), which critically influence permeability *P*. Here, we further introduce ‘penetration path length’ (*t*) to further regulate solvent diffusion. To achieve a universal SC-GPE design, the following factors must be focused: (1) selecting polymer materials with tailored *f*_polymer_ via polymer-solvent interactions; (2) employing large-volume ether molecules (high *V*^∗^_molecule_) to suppress their shuttling toward cathode; and (3) precisely controlling penetration path length (*t*):


(3) \begin{eqnarray*}
D{\mathrm{\ }} \propto {t}^{ - 1}{\mathrm{\ }} \times {\mathrm{\ exp}}\!\!\left( {\frac{{ - V_{{\rm molecule}}^ * }}{{{f}_{{\rm polymer}}}}} \right).
\end{eqnarray*}

**Figure 6.**
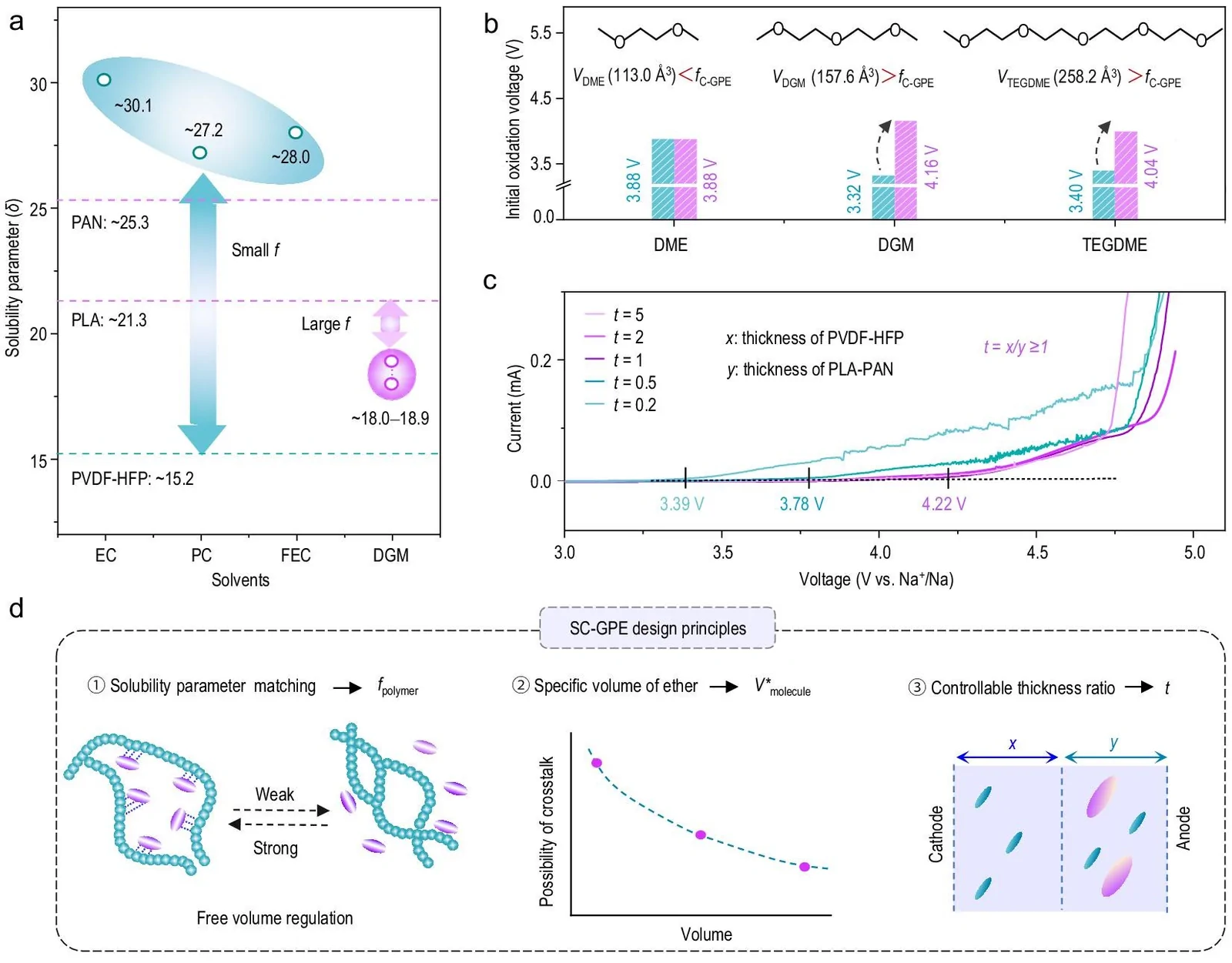
Generalizable design principles of step-channel GPE for scalable SSSBs. (a) The solubility parameter of the polymer and solvent. (b) Size and (c) thickness effects on SC-GPE’s stability. (d) Design principles of the SC-GPE.

The tailored free volume in GPEs is enabled by controlling polymer-solvent interactions, which is based on the chemical stability of polymer matrix. In this study, varying polymers, including PAN, PLA, poly(ethylene oxide) (PEO), nylon-6, polyvinylpyrrolidone (PVP), polyethersulfone (PES), cellulose acetate (CA), PES-PVDF-HFP, and PLA-PAN, were systematically evaluated. Chemically unstable polymers (e.g. CA and PEO) shrink/dissolve due to strong hydrogen bonding (O–H/C≡N) (Fig. S33). PVDF-HFP is selected for the cathode side due to its F-induced oxidation stability, with its highly differentiated solubility parameter with EC/PC/FEC molecules (PVDF-HFP: ∼15.2 vs. EC/PC/FEC: ∼27.2–30.1) (Fig. 6a), the overall thermodynamic mismatch represented by solubility parameters prevents uniform mixing of solvents with polymer chains, leading to the aggregation of PVDF-HFP chains and formation of narrow channels. Conversely, the appropriately matched solubility parameter of PLA-PAN-DGM promotes solvent intercalation, expanding polymer channels. Thus, controllable polymer-solvent matching could achieve a spatially step-channel GPE design.

The solvent-sieving function of SC-GPE was examined through its interfacial stability when accommodating linear ether molecules with different *V*^∗^_molecule_ (Fig. 6b). Specifically, small-sized dimethoxyethane (DME, 113.0 Å^3^) and larger-sized tetraethylene glycol dimethyl ether (TEGDME, 258.2 Å^3^) were introduced. Despite the presence of the high-voltage-stable PVDF-HFP framework, the free volume in the C-GPE (155.8 Å^3^) is insufficient to block DME’s (113.0 Å^3^) shuttling. As a result, the DME-involved A-GPE (3.88 V) and SC-GPE exhibit similar oxidation behavior at 3.88 V. In contrast, the oxidation voltages of the SC-GPE involving DGM and TEGDME are extended to 4.16 V and 4.04 V, respectively, with no decomposition tendency observed at 3.38 V and 3.54 V (Fig. S34a–c). These results demonstrate that only when the *V*^∗^_molecule_ exceeds *f*_polymer_ can a high-voltage-stable SC-GPE be achieved.

Another critical factor is the solvent diffusion distance ratio (*t* = *x/y*), which also significantly affects the interfacial stability of the SC-GPE. Systematic evaluation of SC-GPEs with varying thickness ratios between C-GPE (PVDF-HFP, *x*) and A-GPE (PLA-PAN, *y*) reveals a distinct decomposition behavior—bilayer configurations with *t* < 1 exhibit insufficient oxidative stability, initiating decomposition at 3.39–3.66 V due to DGM crossover. In contrast, configurations with *t* ≥ 1 demonstrate complete suppression of DGM penetration up to 4.0 V, establishing *t* ≥ 1 as one of the critical design criteria (Fig. 6c, values derived from voltage at 0.005 mA). The reproducibility of this thickness-dependent sieving effect is further confirmed through controlled LSV experiments at the limiting condition (*t* = 1, Fig. S35), this thickness-path length relationship provides a scientific principle for precisely tuning interfacial molecular transport.

As demonstrated above, three critical factors govern the design of compatible SC-GPEs: free volume engineering, ether molecular size screening, and penetration path control (Fig. 6d), specifically: (1) *f*_C-GPE_ < *f*_A-GPE_, (2) *V*^∗^_molecule_ > *f*_polymer_, and (3) *t* ≥ 1. Furthermore, we changed a series of ester/ether electrolyte formulations in SC-GPE, the resulting SSSBs can cycle stably (Fig. S36), validating structure’s effectiveness. This design principles offer valuable guidance for subsequent research on interfacial electro-chemo-mechanical adaptability and high-energy-density solid-state batteries (including sodium-based, lithium-based and magnesium-based, etc.).

## CONCLUSION

In summary, we engineered a well-designed step-channel GPE that successfully addresses the intrinsic interfacial challenges of (electro)-chemical instability and mechanical contact loss in highly reactive SSSBs. The step-channel is constructed through two distinct polymer-solvent bindings, connected by shared acrylate monomers and C=O/C–F dipole adsorption: rigid PVDF-HFP tightly interacting with cyclic EC/PC/FEC solvents builds solvent-occupied narrow channels (155.8 Å^3^), while flexible PLA-PAN incorporating linear DGM solvents are more mobile, creating wider channels (173.5 Å^3^). This engineered architecture design imposes bidirectional constraints on solvent migration. The narrow channel selectively blocks oxidation-unstable and long-chain DGM solvents (157.6 Å^3^) from contacting high-voltage cathodes, while the wide channel limits the migration of reduction-unstable EC/PC/FEC into active Na anode. The solvent-restricted SC-GPE across a wide voltage window forms dense, NaF/NaCl-rich CEI (from esters) and NaF/Na_2_S/Na_2_SO_3_-rich SEI (from ethers), effectively passivating electrodes and mitigating side reactions. Mechanistically, the strong local dipole between PVDF-HFP polymer and EC/PC/FEC solvents mitigate solvent drag and impart mechanical elasticity, achieving close contact with high-loading cathodes. Conversely, the weak binding of PLA-PAN polymer and DGM solvents promotes viscous deformation, enhancing interfacial Na adhesion. As a result, the Na||NFS pouch cell paired with SC-GPE achieves an energy density of 191.3 Wh kg^−1^ (NFS loading: 18.7 mg cm^−2^) and stable cycling (100 cycles, 100% retention). Furthermore, with the increase of cathode loading (NVP loading: 34.1 mg cm^−2^), the Na||NVP full cell achieves a high energy density of 345.6 Wh kg^−1^, surpassing existing systems. The superior performance stems from the (electro)-chemically and mechanically compatible design of the SC-GPE, as revealed by the P2D model, to effectively mitigate active material loss caused by interfacial contact failure or bubble blockage during cycling (SC-GPE: 3.544 × 10^−4^ vs. C-GPE: 6.832 × 10^−4^ mm^−3^). The abuse testing (needle puncture, short circuit, cutting) of Na||NVP pouch cells verified the high safety of SC-GPE assembled SSSBs. Cathode compatibility is broadly demonstrated with Na_*x*_Fe(P_2_O_7_)_5_·*x*H_2_O, NVP, and Na_0.72_Ni_0.32_Mn_0.68_O_2_. By integrating polymer design, electrolyte engineering, and mechanical compatibility, we first introduce generalizable design principles for step-channel GPE that address key interfacial barriers toward practical, high-performance solid-state batteries.

## Supplementary Material

nwag291_Supplemental_File
