# Supplementary material for: A step-channel gel polymer electrolyte for solid-state sodium batteries
Source: Natl Sci Rev. 2026 May 26;13(17):nwag291. doi: 10.1093/nsr/nwag291 (PMC13552063; doi:10.1093/nsr/nwag291)
Supplement: Supplementary file 1 [file nwag291_supplemental_file.pdf]

Supporting Information

A step-channel gel polymer electrolyte for solid-state sodium batteries

Xiaoniu Guo[1,†], Shuai Guo[1,†], Shaohua Ge[1,†], Longfei Wen[1], Zhichao Gong[2], Yi Yang[1], Junyu Li[1], Ruixue Wang[1], Zhengkun Xie[1], Jiyu Zhang[1], Jun Luo[1], Jianqiang Kang[2], Lingfei Zhao[3], Jun Lu[4,*], Junhua Shao[5] and Weihua Chen[1,*]

[1]College of Chemistry, Zhengzhou University, Zhengzhou 450001, China;

[2]Hubei Key Laboratory of Advanced Technology for Automotive Components, Wuhan University of Technology, Wuhan 430070, China;

[3]Centre for Clean Energy Technology, School of Mathematical and Physical Sciences, University of Technology Sydney, Sydney NSW 2007, Australia;

[4]College of Chemical and Biological Engineering, Zhejiang University, Hangzhou 310027, China and [5]Henan Finelyte New Energy Technology Co., Ltd., Jiaozuo 454000, China

***Corresponding authors.** E-mails: chenweih@zzu.edu.cn; junzoelu@zju.edu.cn

[†]Equally contributed to this work.

1. Materials and methods

1.1 Preparation of PLA-PAN and PVDF-HFP matrix

PLA-PAN matrix was prepared by electrospinning. First, 0.9548 g PLA particles and 0.4092 g PAN powder were dissolved in DMF at 60 ^{o}C, and magnetically stirred for 2 h to obtain a uniform spinning solution. Next, the PLA-PAN solution was injected into a 10 mL syringe with an inner diameter of 0.84 mm, with a spinning voltage of 21 kV, and the spinning rate was 1.0 mL h^{-1}. Finally, the PLA-PAN matrix with aluminum (Al) foil was dried in a vacuum at 50 ^{o}C for 12 h. The PVDF-HFP, sourced from Energy Chemical, was prepared using the aforementioned procedure, the difference is that the stirring temperature was maintained at room temperature, the spinning rate was set to 0.8 mL h^{-1}, and the polymer concentration was 25 wt%.

1.2 Preparation of C-GPE, A-GPE, and SC-GPE

First, to prepare the SC-GPE consisting of C-GPE and A-GPE, the corresponding precursor solutions were precisely prepared. The 0.2 g TFMA (M_w=168.11, MACKLIN reagent), 0.2 g PETEA (M_w=352.34, MACKLIN reagent), and 0.012 g photoinitiator were precisely introduced into 3.6 g 1M $NaClO_4$ EC PC 5wt% FEC or 1M $NaCF_3SO_3$ DGM. The mixture was subjected to continuous stirring at an ambient temperature for 10 min. After that, the preparation begins with 40 μL high-voltage stable precursor dropped on a PTFE plate, subsequently taken up by electrospinning PVDF-HFP matrix, after 4 minutes of UV irradiation, the C-GPE was successfully prepared. After that, the PLA-PAN matrix absorbing the ether precursor was solidified on C-GPE and finally formed the SC-GPE. All preparation processes are operated under an Ar atmosphere.

2. Materials and instruments

2.1 Materials and characterizations

The morphology and structure of the SC-GPE were obtained by scanning electron microscope (SEM, ZEISS Merlin Compact). The chemical bonds and phase transformation were recorded by a Fourier transform infrared spectroscopy (FTIR, Nicolet iS50). The solvation structure of SC-GPE was determined by the 532 nm Raman spectrometer (LabRAM HR Evolution). The CEI was detected via X-ray photoelectron spectroscopy (XPS, Escalab250Xi), and the SEI on Na surface was detected by Thermo Fisher Scientific K-Alpha, all sample preparation processes operated under an Ar atmosphere. The stress-strain response was measured by a tensile machine (INSTRON-5967) with a stretching rate of 10 mm min^{-1}. The rheological measurements were performed using oscillatory shear rheology (TA/waters HR-20) with parallel-plate geometry. The oscillatory shear experiments were carried out at a strain of γ = 2%, a shear rate of ω = 0.1~100 rad s^{-1}, and at ambient temperature. The XRD (PANalytical X'Pert PRO) was used to characterize the sample's crystallinity and phase transformation with Cu Kα radiation (λ=1.54178 Å), ranging from 10° to 55° with a scan rate of 5° min^{-1}. The free volume of PVDF-HFP and PLA-PAN polymers was measured via Positron Annihilation Lifetime Spectroscopy (PALS, AMETEK PLS-SYSTEM)

2.2 Cryo-transmission electron microscope (Cryo-TEM)

Cryo-TEM was performed using FEI Glacios (USA), which operated at 200 kV and was

equipped with an automatic injection system of frozen samples. The preparation procedures for CEI and SEI samples are as following:

For the conventional particulate NFS cathode, the CEI preparation procedure is as follows: First, Na||NFS cells are assembled. After cycling to a specific state, the NFS particles are carefully scraped off the Al current collector and dispersed in PC solvent. Three drops of the dispersion are then dropped onto a copper grid. The grid is left to stand in a glovebox until the PC solvent completely evaporates, yielding a dry CEI sample for characterization.

For the bulk sodium metal anode, the SEI preparation procedure is as follows: In contrast to the CEI preparation, Na||Cu cells are assembled. Sodium metal is deposited on the Cu foil at a specific capacity (0.5 mAh cm^{-2}). The Na||Cu cell is then disassembled, and the Cu foil with deposited sodium is cut into small pieces and dispersed in a sample vial containing PC solvent. The vial is placed in an ultrasonic cleaner for 30 minutes to ensure that the SEI-containing sodium metal detaches from the Cu foil and disperses into the PC solvent. After sonication, the vial is transferred into a glovebox, and three drops of the dispersion are dropped onto a copper grid and dried inside the glovebox. It is worth noting that all sample handling steps are performed under Ar atmosphere.

2.3 In-situ differential electrochemical mass spectrometry (In-situ DEMS)

In-situ DEMS experiments were conducted in a custom-made cell obtained from Beijing Scistar Technology Co. Ltd., which was assembled in an argon-filled glove box. Pre-dehydrated helium (99.999%) was employed as the carrier gas. A high flow rate purged the pipeline to remove the air. Then, a gas controller controlled the flow rate at 1 mL min^{-1}. The gas products during the charge and discharge process were carried through an intelligent low-temperature thermostatic bath to condense the electrolyte vapor before entering the mass spectrometer (HPR-20 DEMS, Hiden Analytical).

2.4 In-situ optical microscopy

The Na deposition process was in situ observed by Na||Na symmetrical cells under optical microscopy. The Na metal foils were cut into electrodes of the same size, and the SC-GPE was sandwiched between the two Na electrodes, and the current density was 1.0 mA/cm^2.

3. Battery assembly and electrochemical measurements

3.1 Electrode preparation and battery assembly

$Na_{2.466}Fe_{1.724}Mg_{0.043}(SO_4)_3$ (NFS) was synthesized based on the previously reported work [S1]. $Na_3V_2(PO_4)_3$ (NVP, ≥99.5%) was purchased from Shenzhen Kejing Zhida Technology Co., Ltd, $Na_{0.72}Ni_{0.32}Mn_{0.68}O_2$ (NNM, ≥99.0%) was provided by Shenzhen Paragonage Technology Co, Ltd. $Na_xFe(P_2O_7)_5{\cdot}xH_2O$ (NFPP), polyvinylidene fluoride (PVDF, ≥99.5%, molecular weight: 1 million) and Super P were purchased from Canrd Technology Co. Ltd. The NVP, NNM and NFPP electrodes were mixed with Super P and PVDF solution in a weight ratio of 8:1:1 to form a series of homogeneous slurry mixtures, which were coated onto clean Al foil and vacuum dried at 120 ^oC for 24 h, respectively. The NFS electrode was mixed with Super P and PVDF in a weight ratio of 7:2:1. All the electrodes were coated on one side. The high-loading (>10 mg cm^{-2}) NFS and NVP (>20 mg cm^{-2}) electrodes was prepared with a ratio of 8:1:1 and 92:4:4, the thickness of Na foil in in the coin cell is ~150 μm, while the thickness of Na foil used in the pouch cell is ~70 μm (purchased

from Jiaxing Changhao New Material Technology Co., LTD). After assembling 2025-types Na||NFS, Na||NVP, Na||NNM, Na||NFPP cells in a high-purity argon filled glove box (Mikrouna), they are charged and discharged within voltage ranges of 2.5-4.0 V, 2.0-4.2 V, 1.5-4.0 V, and 2.0-4.5 V.

3.2 Measurements of ionic conductivity (σ)

The ionic conductivities of GPE were tested by electrochemical impedance spectroscopy in stainless steel||stainless steel symmetric cells over a frequency range of 0.1 to 100 kHz (Metrohm workstation). The ionic conductivities of GPE were calculated by the following Equation (1):

$$\sigma = \frac{L}{R \times A} \quad (1)$$

where L is the thickness of the GPE, R is the resistance of the bulk GPE, and A is the effective contacting area between the GPE and the stainless-steel sheet.

3.3 Measurements of Na^+ transference number (t_{Na+})

According to the Bruce-Vincent method, DC polarization measurements were conducted with a potential of ΔV=10 mV in the Na||Na symmetric cells until the current reached a steady state. The ion transference number using a potentiostatic polarization method, as shown in the following Equation (2):

$$t=\frac{I_{ss}(\Delta V-I_0R_0)}{I_0(\Delta V-I_{ss}R_{ss})} \quad (2)$$

where ΔV is the voltage polarization applied, I_{ss} and R_{ss} are the steady-state current and resistance, respectively, and I_0 and R_0 are the initial current and resistance, respectively.

3.4 Measurements of Na^+ conductivity (σ_{Na+})

$$\sigma_{Na+}=\sigma\times t_{Na+} \quad (3)$$

3.5 Linear sweep voltammetry (LSV)

The oxidation and reduction stability of the GPE were measured by Na||stainless steel cells or Na||NFS cells using the linear sweep voltammetry (LSV) method on a Metrohm workstation at a scan rate of 1 mV s^{-1} at room temperature.

4. Calculation methods

4.1 Density functional theory method (DFT)

All DFT calculations were performed using the Gaussian 16 program package. All geometry optimizations were performed at Becke's three-parameter hybrid method using the Lee–Yang–Parr correlation functional (B3LYP) method at 6–31G basis set. The geometrically optimized single-point energy was calculated using the 6-311++G (d, p) basis set. The binding energies were obtained via single-point energy calculations. Gaussian 09W was used to simulate the interaction between PAN, PLA, PVDF-HFP, and solvents in the electrolyte, and the binding energy was calculated by DFT. To make the calculation affordable, the monomers of PAN, PLA, and PVDF-HFP represent polymers. The binding energy between solvent and units was calculated by $E_b = E_{complex}–E_{unit}–E_{solvent}$ (where $E_{complex}$ is the energy of the bound complex; E_{unit} is the energy of the monomers; $E_{solvent}$ is the energy of the solvent, respectively). Note that all optimizations start with multiple initial guesses of the structure, and the most stable guesses are saved. The orbital composition of solvent size,

polymer bond angle, polymer bond length, and dipole moment were analyzed based on the optimized molecular structure above.

4.2 Molecular dynamics (MD) simulations

(1) The diffusion coefficients of film-forming molecules in A-GPE: Electrolyte models (60×60×60 Å^3) were constructed to model the Na^+, $CF_3SO_3^-$, DGM, PAN, and PLA. The electrolyte model contains 10 Na^+, 10 $CF_3SO_3^-$, 74 DGM, 267 PAN, and 642 PLA. (2) The diffusion coefficients of film-forming molecules in C-GPE with a small amount of ester: Electrolyte models (65×65×65 Å^3) were constructed to model the Na^+, ClO_4^-, EC, PC, FEC, and PVDF-HFP. The electrolyte model contains 20 Na^+, 20 ClO_4^-, 150 EC, 118 PC, 12 FEC, 770 PVDF-HFP, and 100 DGM. (3) The diffusion coefficients of oxidation-resistant molecules in A-GPE with a small amount of ether: Electrolyte models (65×65×65 Å^3) were constructed to model the Na^+, $CF_3SO_3^-$, DGM, EC, PC, FEC, PAN, and PLA. The electrolyte model contains 10 Na^+, 10 $CF_3SO_3^-$, 74 DGM, 75 EC, 59 PC, 6 FEC, 267 PAN, and 642 PLA.

The atomistic simulation studies (universal) force field was chosen for all the MD calculations. Besides, the time step was set to be 1 fs. The electrolyte systems were equilibrated in the isothermal-isobaric ensemble (constant NPT) using the Berendsen barostat to maintain the pressure of 0.0001 GPa with a decay constant of 0.1 ps for 1000 ps. The temperature was set to 298 K with the Nose thermostat. Then, the production run of 1000 ps was obtained in the canonical ensemble (NVT) at 298 K. Equilibrium states of the electrolyte systems were acquired with a long enough simulation time. The solvent diffusion coefficient was obtained by MD simulations of the optimized model.

4.3 Modeling

This research constructs an electrochemical model of Na||NFS half-cell based on the PyBaMM 24.11 open-source computing platform. This modeling framework uses porous electrode theory to describe the Na^+ migration behavior in the NFS cathode and combines the mass/charge conservation equation of the electrolyte phase with the Butler-Volmer electrode kinetics equation to achieve coupled calculations of the core physical processes. Notably, the model additionally considers the side reactions of gas production. Due to the generation of gas, the Na^+ transport channel is blocked, inducing a loss of effective active area and capacity.

$$Q_{loss,LAM} = \frac{\varepsilon_p \cdot \delta_p \cdot F \cdot c_{s,\max,p} \cdot A_{loss}}{3600} \tag{4}$$

$$dA = \frac{dV}{\delta_p} \tag{5}$$

Where $Q_{loss,\ LAM}$ is the capacity loss, A_{loss} is the loss of effective area in the NFS cathode, ε_p is the thickness of the cathode, δ_p is the volume fraction of NFS, and $C_{s,\ max,\ p}$ is the maximum Na concentration, respectively. By using the above formula, the $Q_{loss,\ LAM,}$ and A_{loss} can be calculated through Equation (4-5). Linear fitting is performed on the volume of failed active materials, where the slope k is the volume V_{loss} of the active substance per cycle. V_{loss} is positively correlated with the gas production rate, which is also positively correlated with the interfacial reaction intensity (Equation (6)).

$$V_{loss} \propto V_{gas} \propto \textit{Rate of interfacial reaction} \tag{6}$$

5. Supplementary Figures and Tables

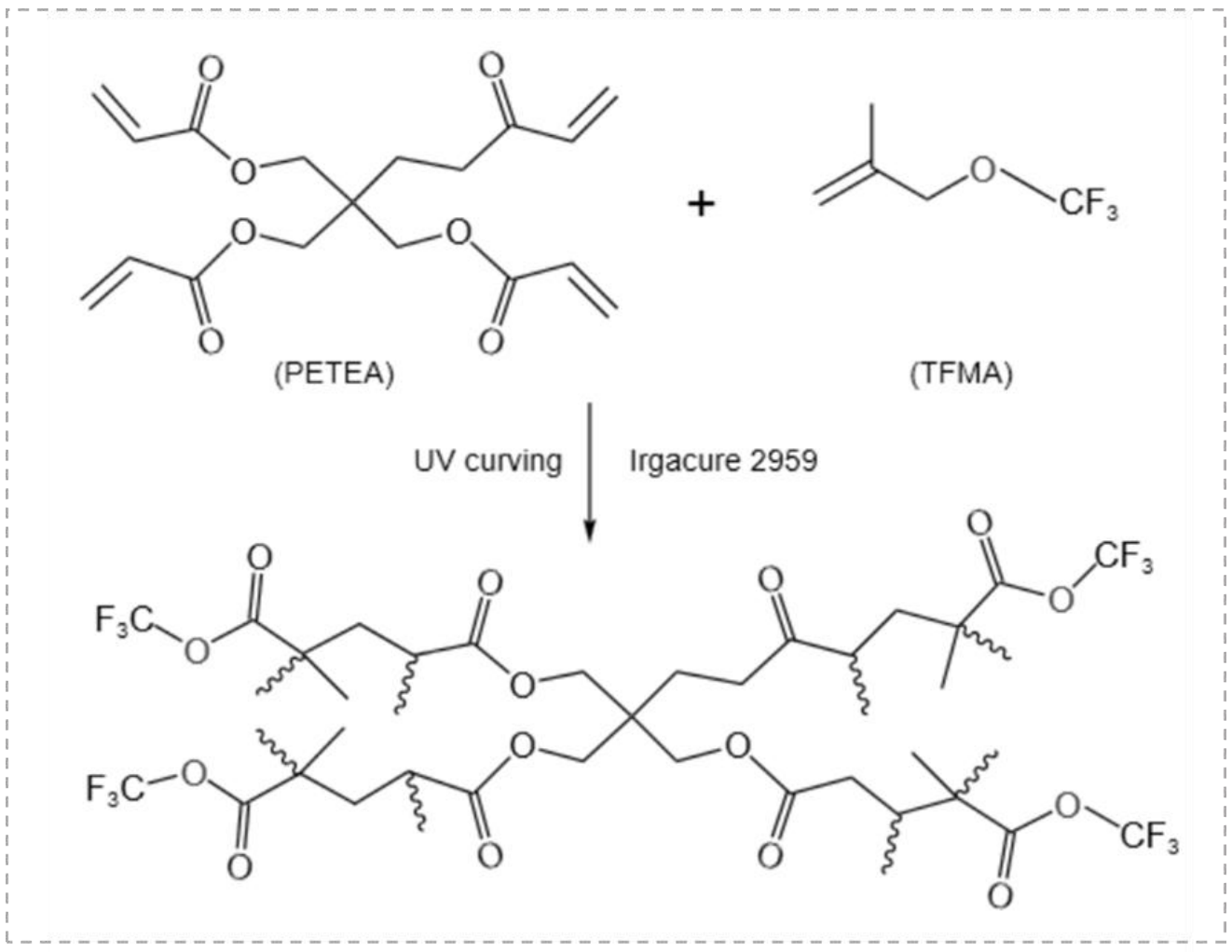

Supplementary Fig. 1 PETEA, TFMA monomers and their crosslinked polymer network.

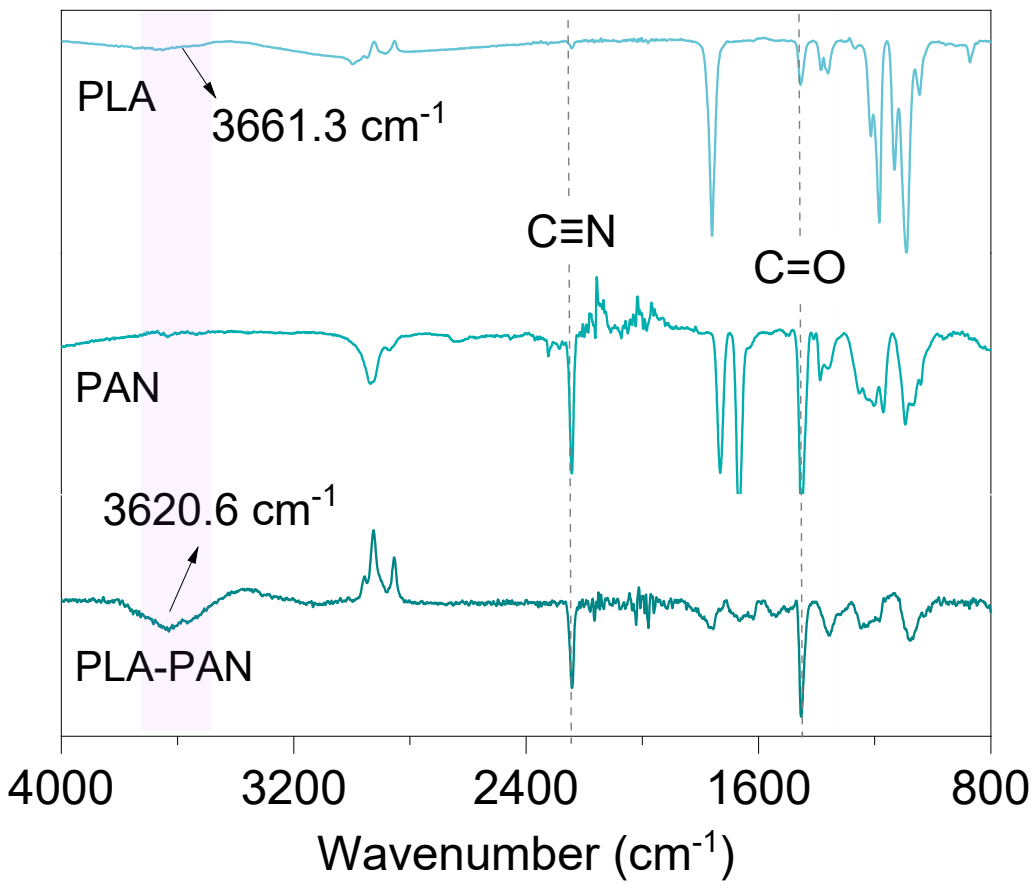

Supplementary Fig. 2 FTIR spectra of PLA, PAN, and PLA-PAN matrix. The formation of -O-H···N interactions between PLA and PAN chains is evidenced by the red-shifted and intensified -OH peaks at 3661.3 cm^{-1} and 3620.6 cm^{-1} in the FTIR spectrum.

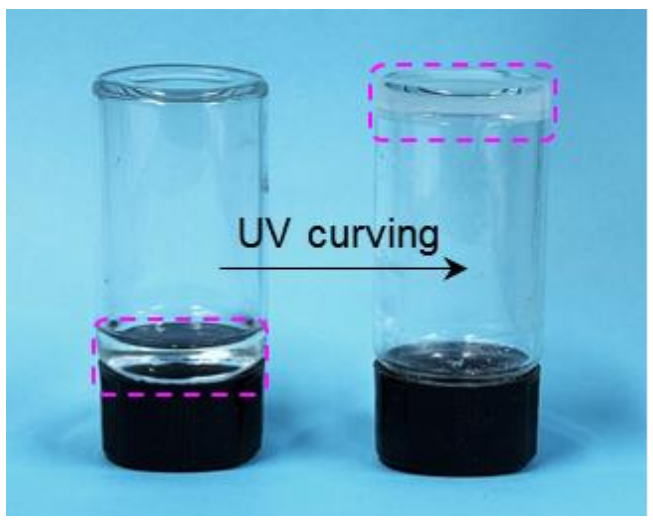

Supplementary Fig. 3 Optical images of precursor and polymerized gel.

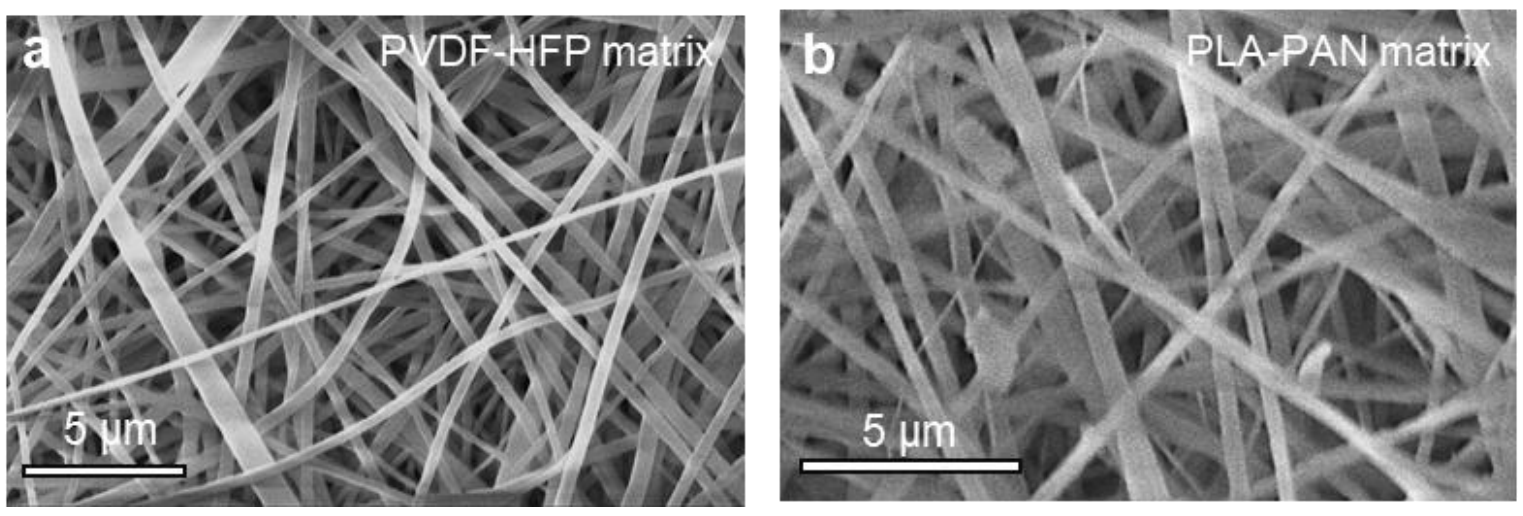

Supplementary Fig. 4 SEM images of (a) PVDF-HFP matrix and (b) PLA-PAN matrix. The PVDF-HFP and PLA-PAN electrospinning frameworks exhibit loose fiber structure, which is beneficial for the subsequent absorption of C-/A-GPEs precursors

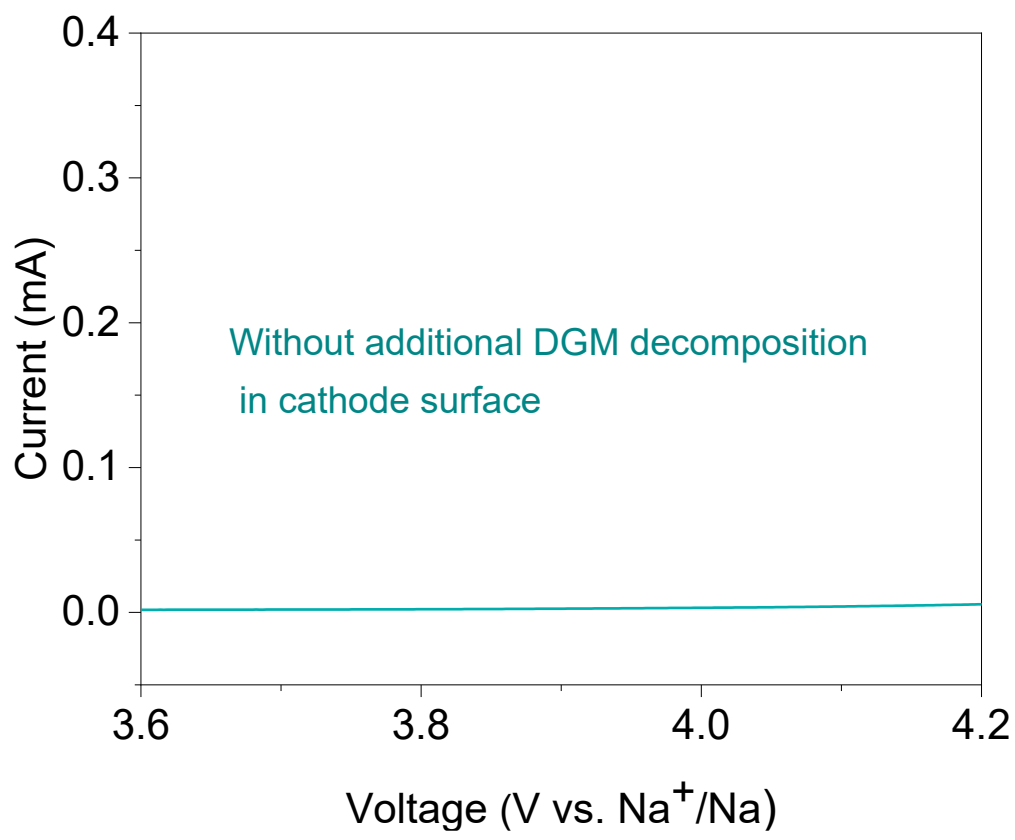

Supplementary Fig. 5 The enlarged LSV curve of the SC-GPE at 3.6-4.2 V.

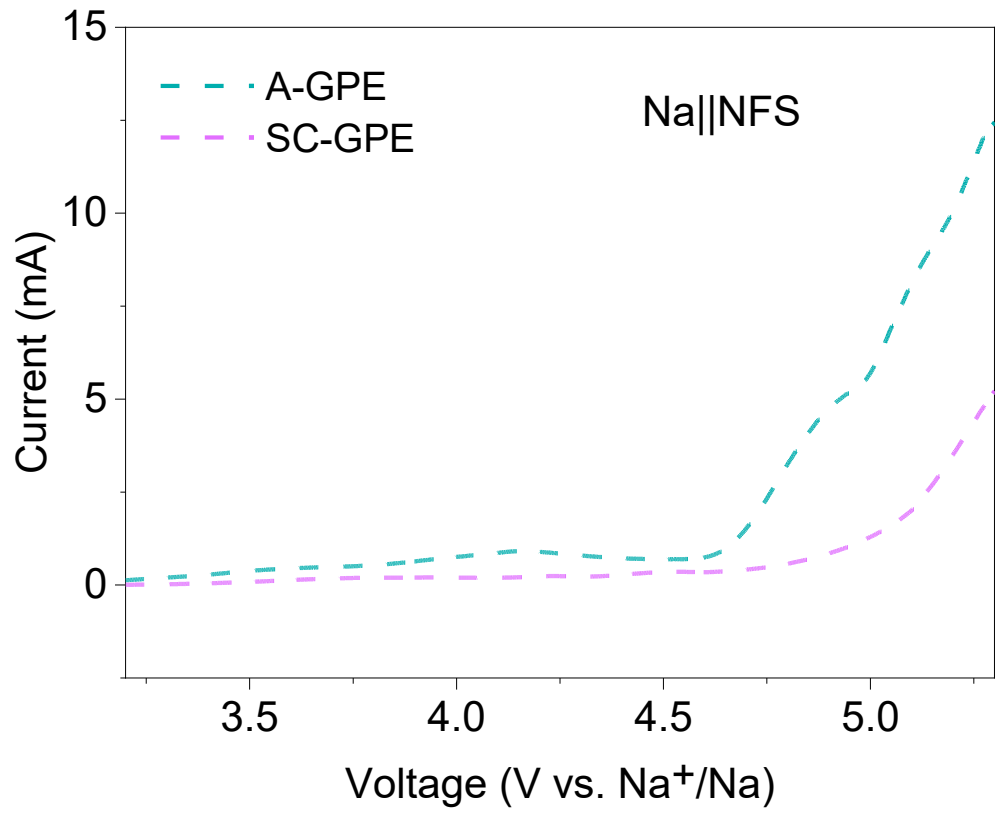

Supplementary Fig. 6 LSV curves of Na||NFS cells using A-GPE and SC-GPE, respectively.

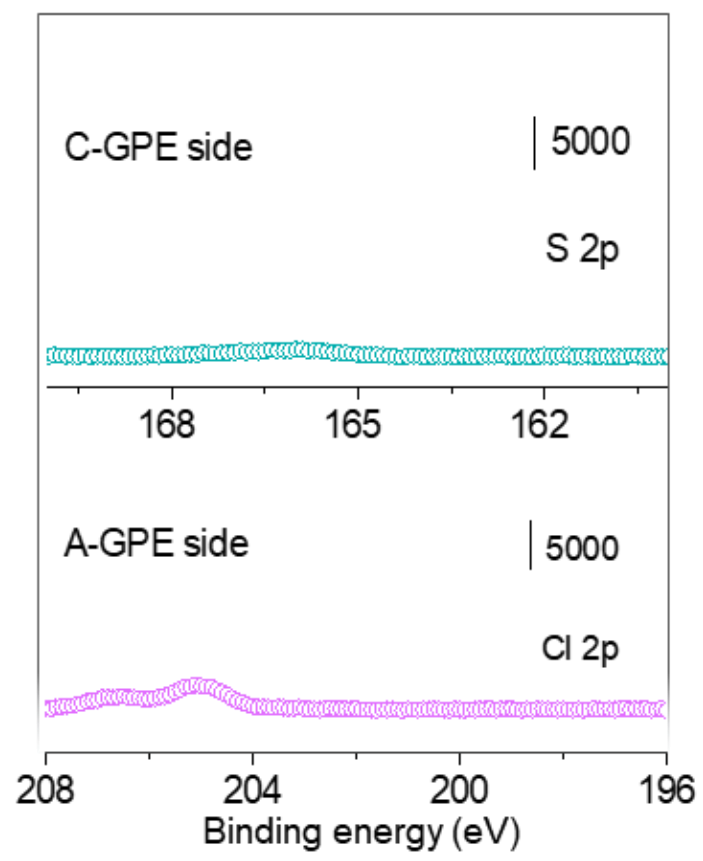

Supplementary Fig. 7 XPS analyses for S2p, Cl2p spectra in C-GPE and A-GPE sides of SC-GPE.

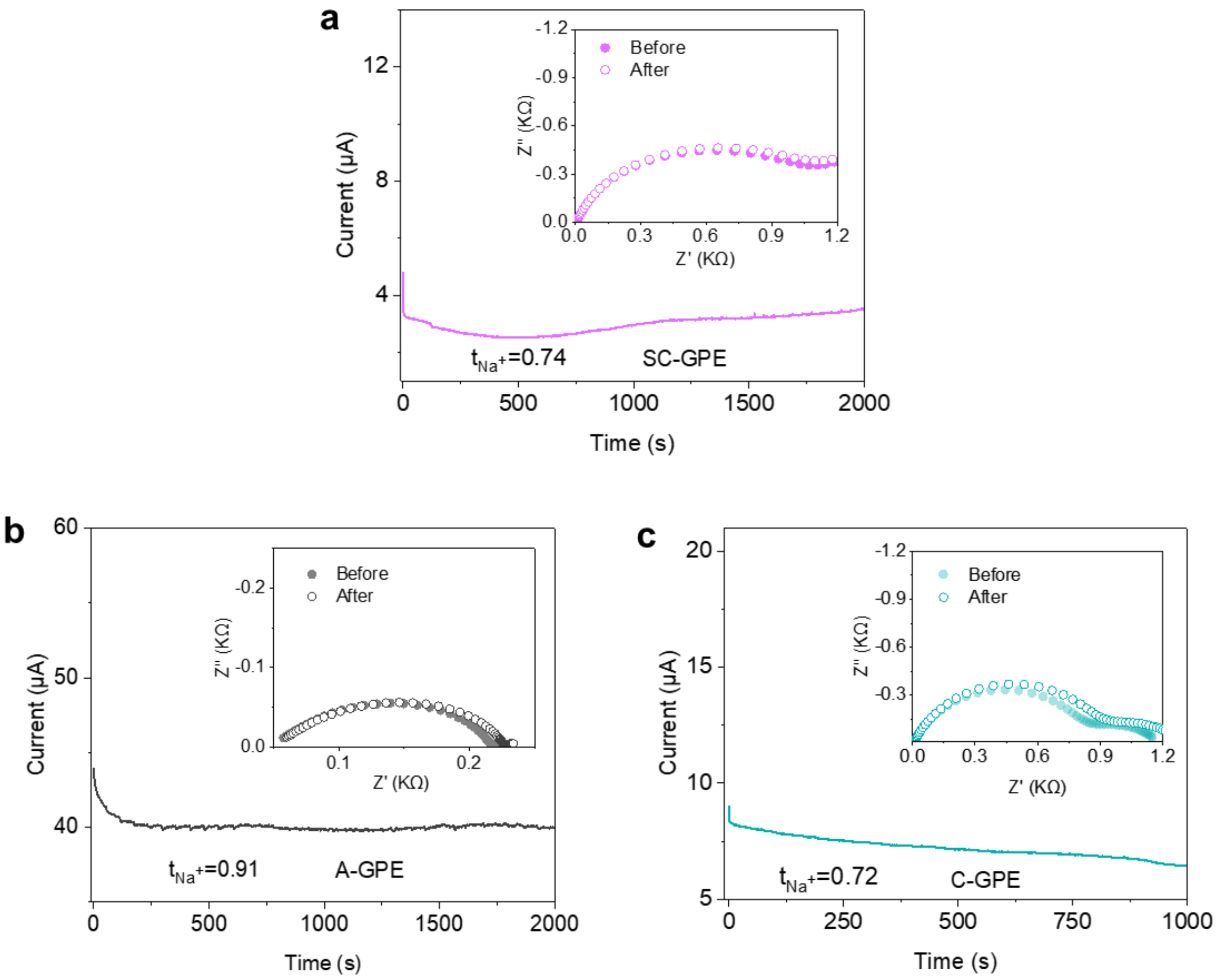

Supplementary Fig. 8 Polarization curves of the Na||Na cells before and after 10 mV polarizing for (a) SC-GPE, (b) A-GPE and (c) C-GPE, the inset is impedance diagrams.

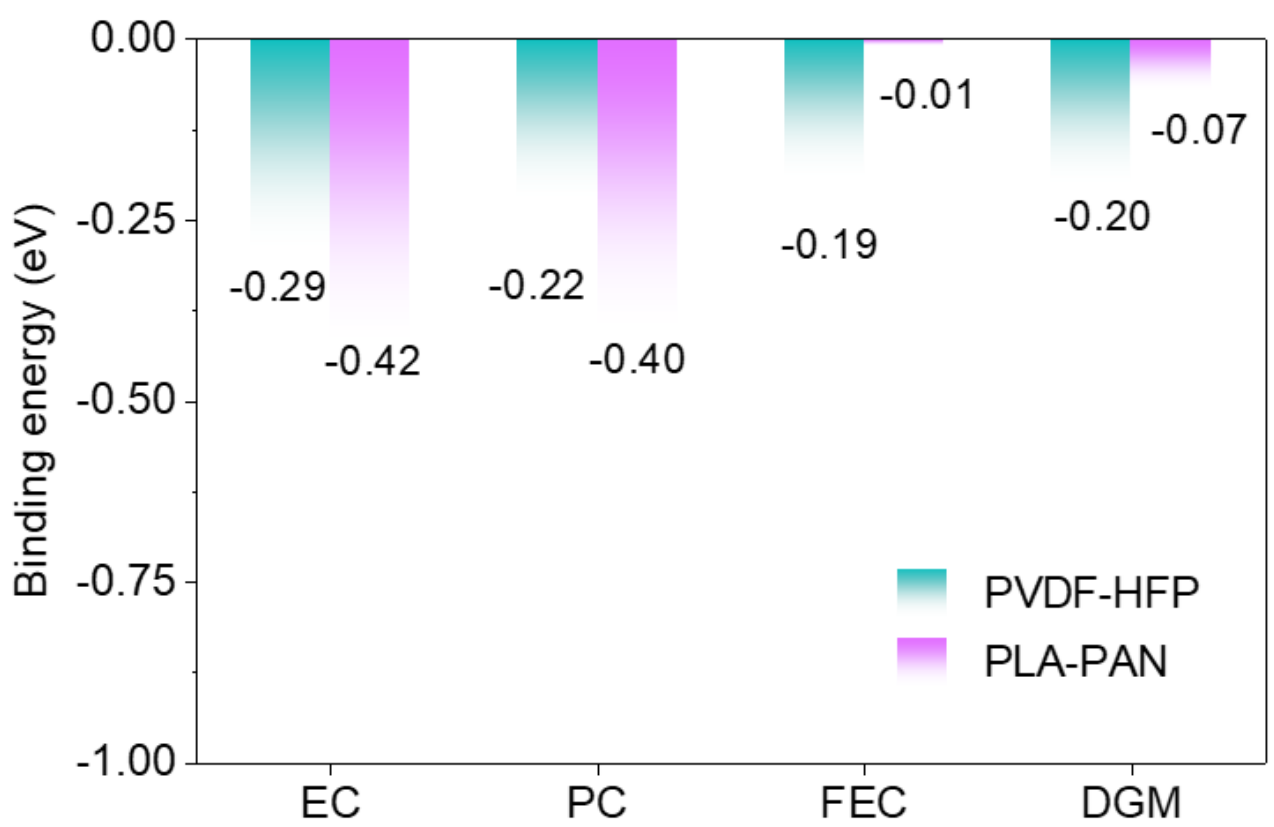

Supplementary Fig. 9 Binding energies between PVDF-HFP, PLA-PAN, and EC, PC, FEC, DGM solvent molecules.

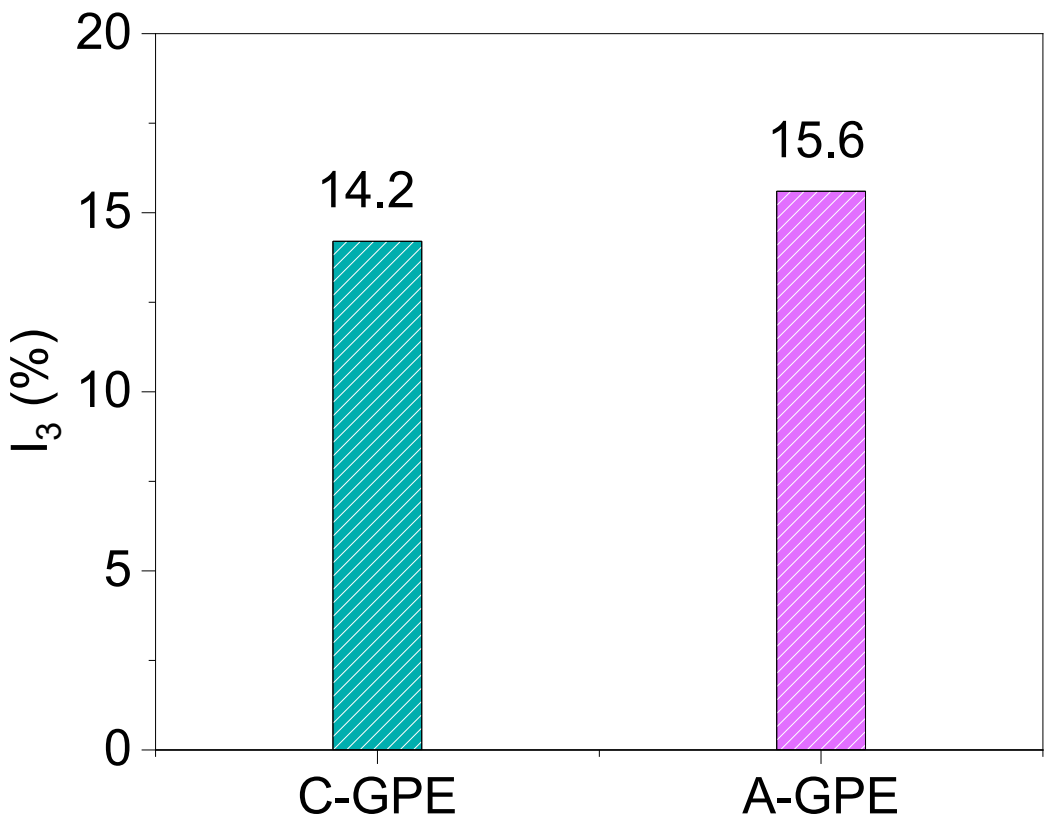

Supplementary Fig. 10 Comparison of I_3 intensity between C-GPE and A-GPE obtained by PALS. The o-Ps intensity (I_3) of C-GPE is only 14.2%, lower than the 15.6% of A-GPE. This may be due to the strong attraction of electron rich ester solvents (C=O) in C-GPE to positrons, inhibiting o-Ps formation and leading to a decrease in I_3.

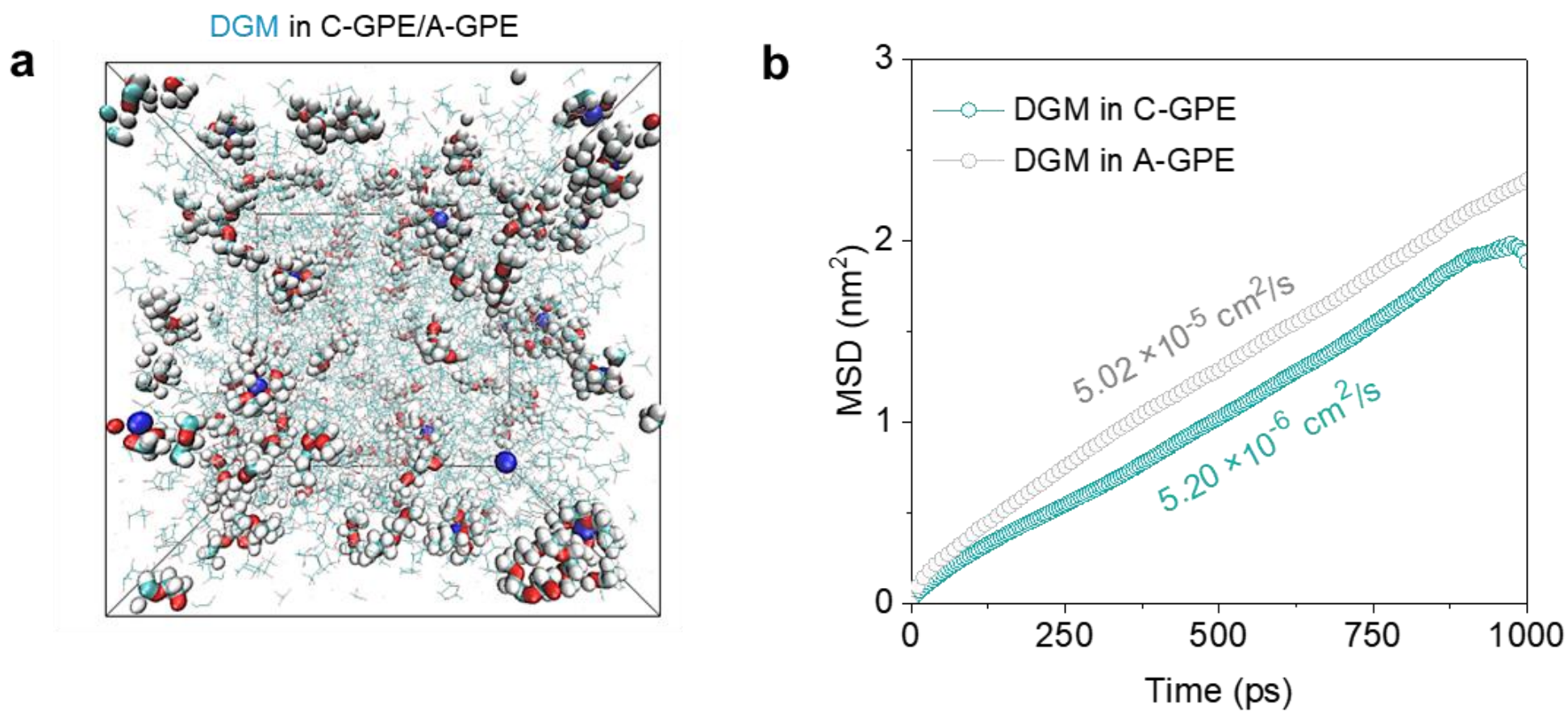

Supplementary Fig. 11 (a) Snapshots of DGM in C-GPE and their respective chemical environments in the equilibrium state. (b) Mean square displacements of DGM in C-GPE and A-GPE.

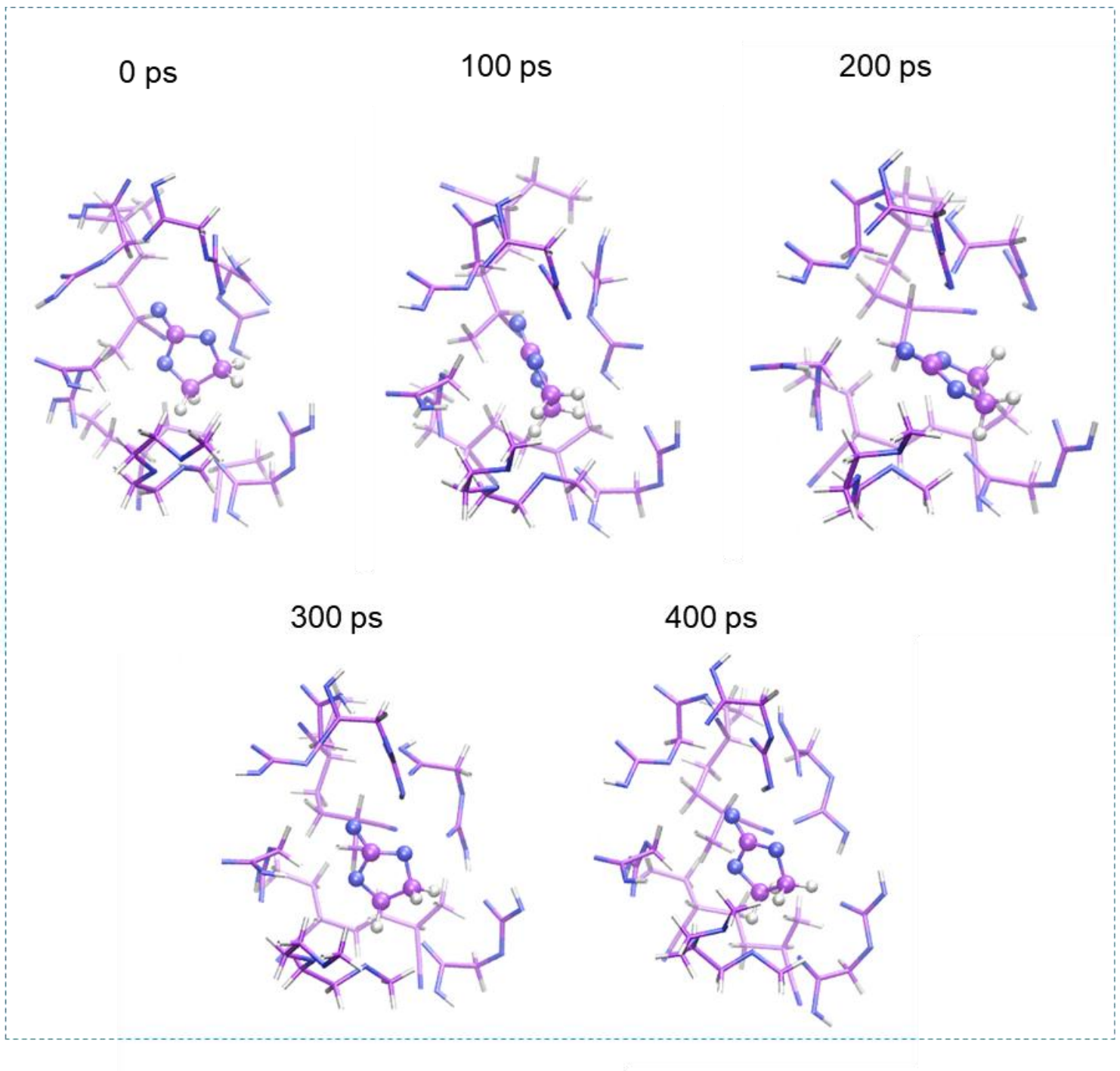

Supplementary Fig. 12 The diffusion of EC molecules within 400ps in A-GPE.

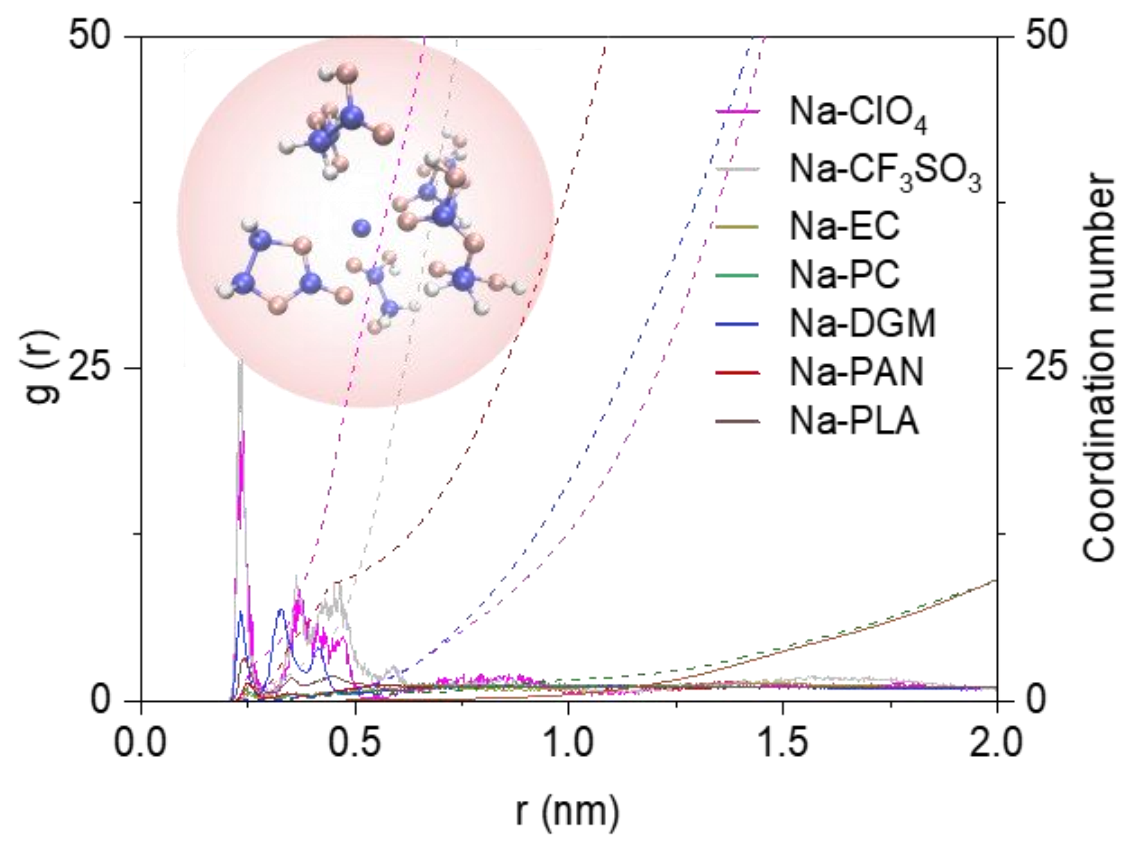

Supplementary Fig. 13 Radical distribution function and the coordination number for SC-GPE.

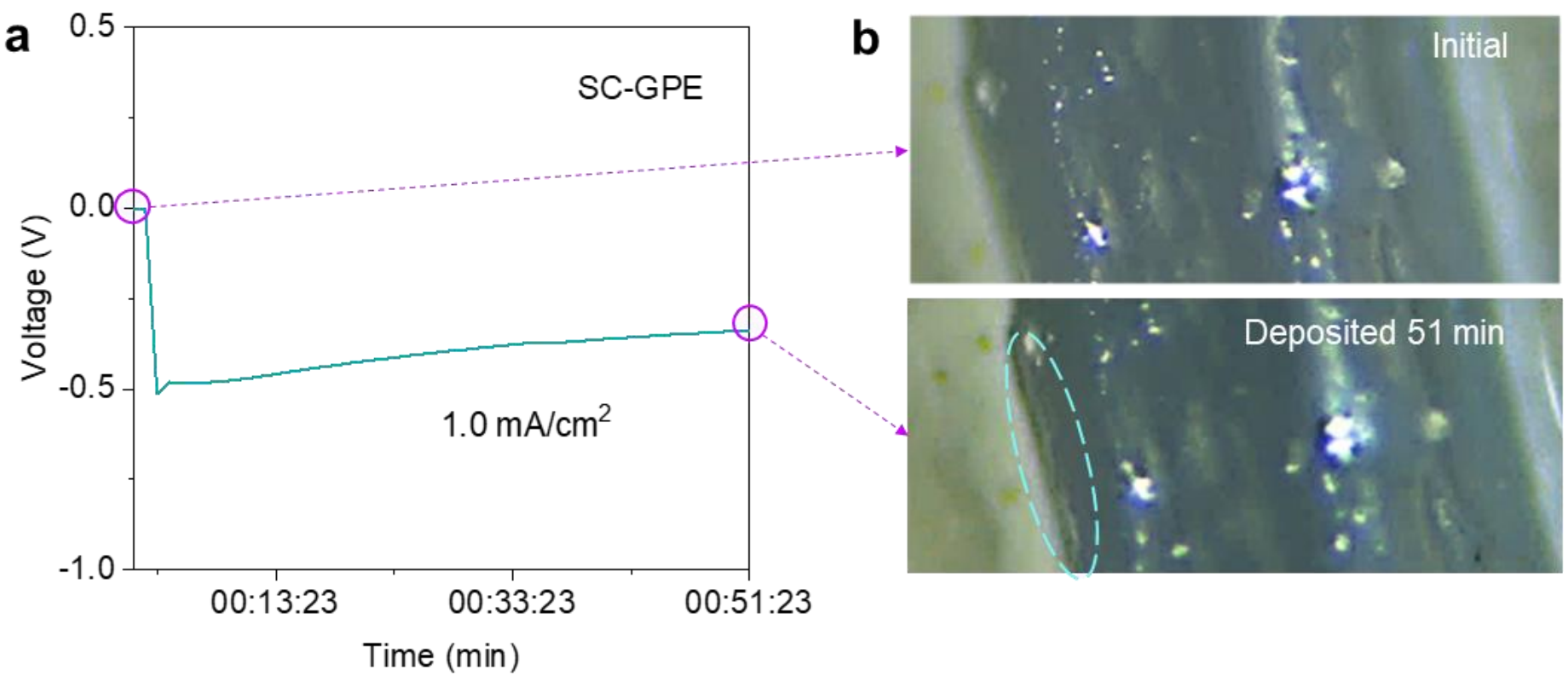

Supplementary Fig. 14 (a) The time-voltage curve of the Na||Na cell using SC-GPE at 1.0 mA/cm^2 and (b) Na deposition images at different times.

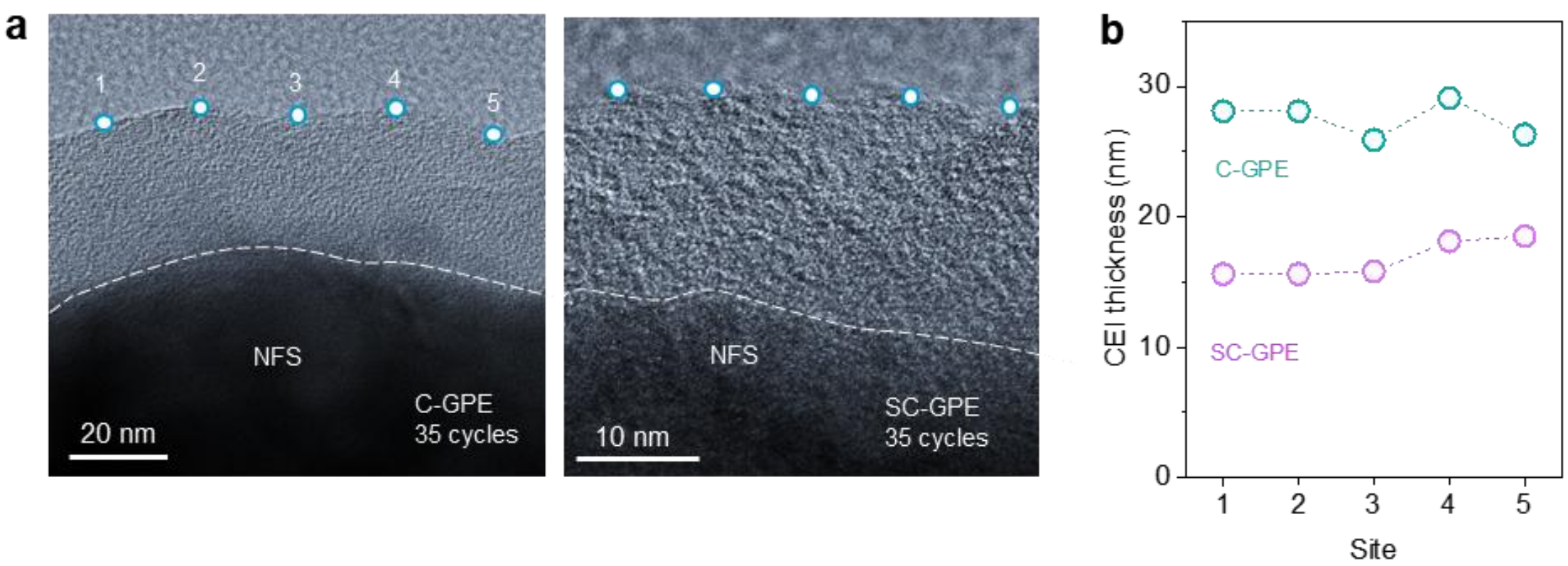

Supplementary Fig. 15 (a) Cryo-TEM images of the SEI formed on NFS cathode after 35 cycles at 0.5 C, 25 °C and (b) their thickness statistics.

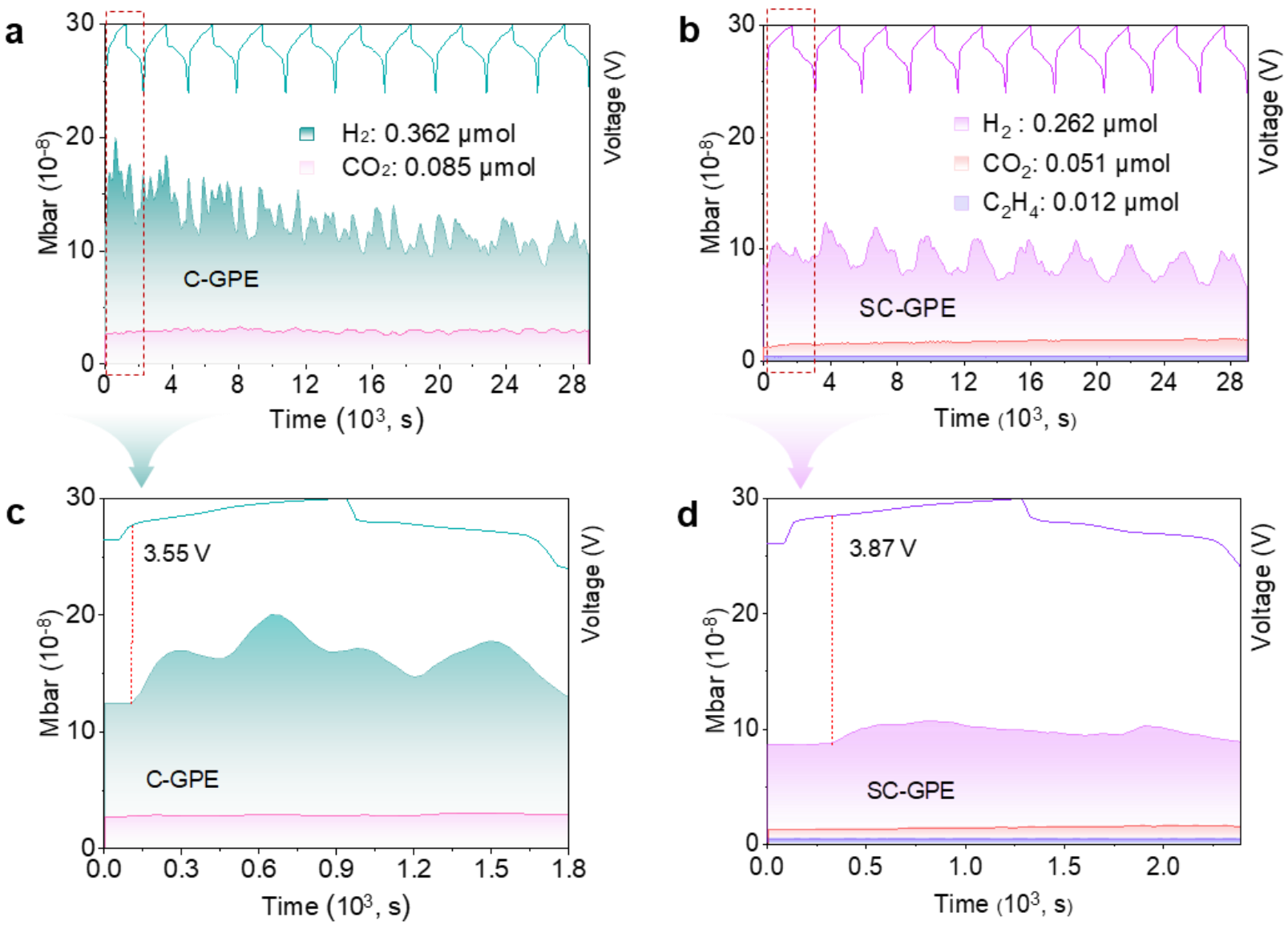

Supplementary Fig. 16 ***In-situ*** **DEMS of Na||NFS cells with (a) C-GPE and (b) SC-GPE during the 10 cycles and (c-d) their local images.**

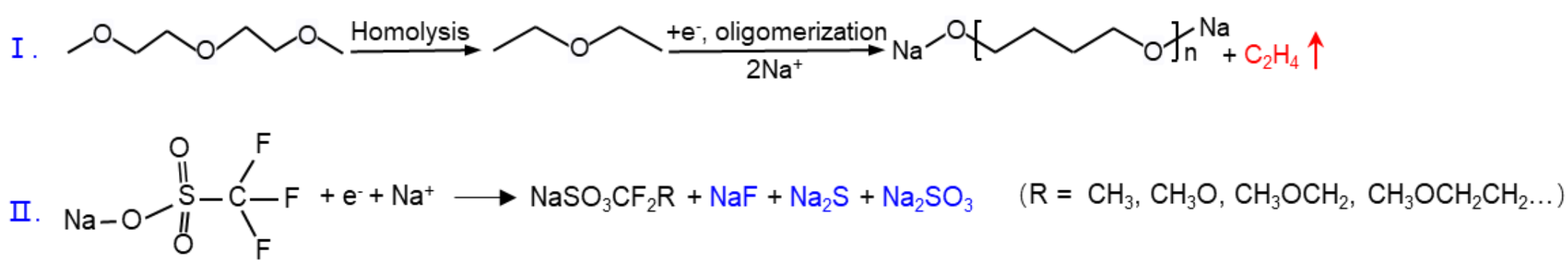

Supplementary Fig. 17 Possible reduction and salt decomposition reactions occurred in Na||NFS cells using C-GPE and SC-GPE.

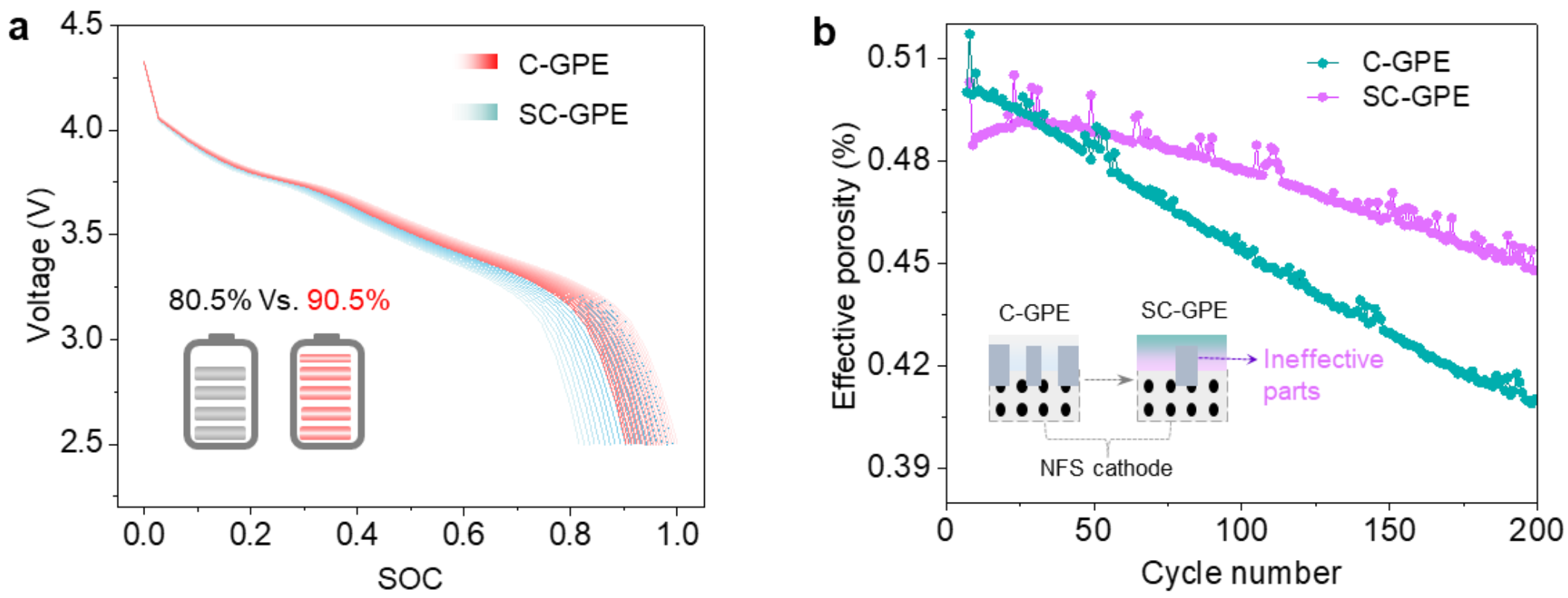

Supplementary Fig. 18 (a) SOC-voltage curves of Na||NFS cells. (b) Effective porosity evolutions of NFS.

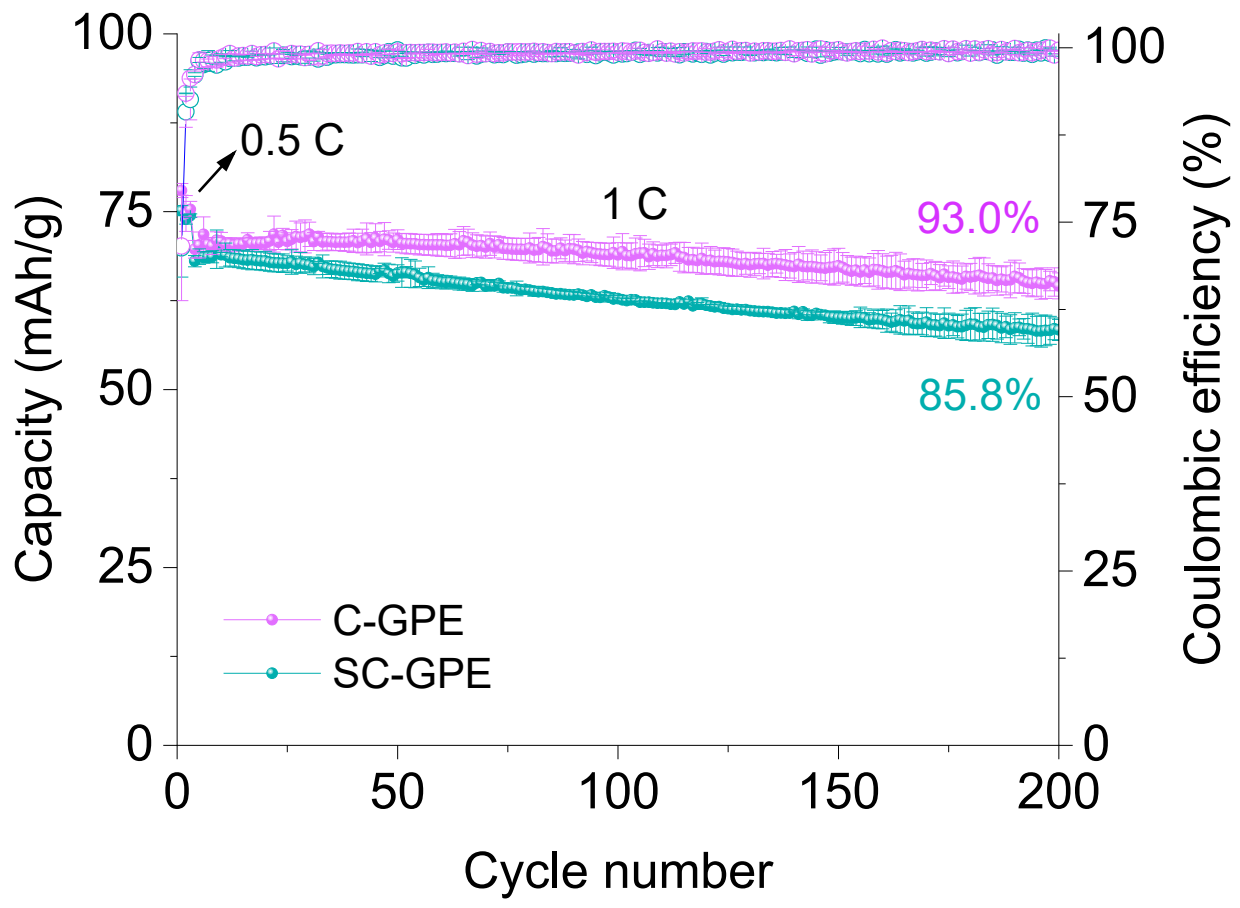

Supplementary Fig. 19 Cycling performance of the Na||NFS cells using C-GPE and SC-GPE after 3 cycles at 0.5 C (1 C=120 mA g^{-1}) and cycling at 1 C, and 25°C, error bars come from three cells.

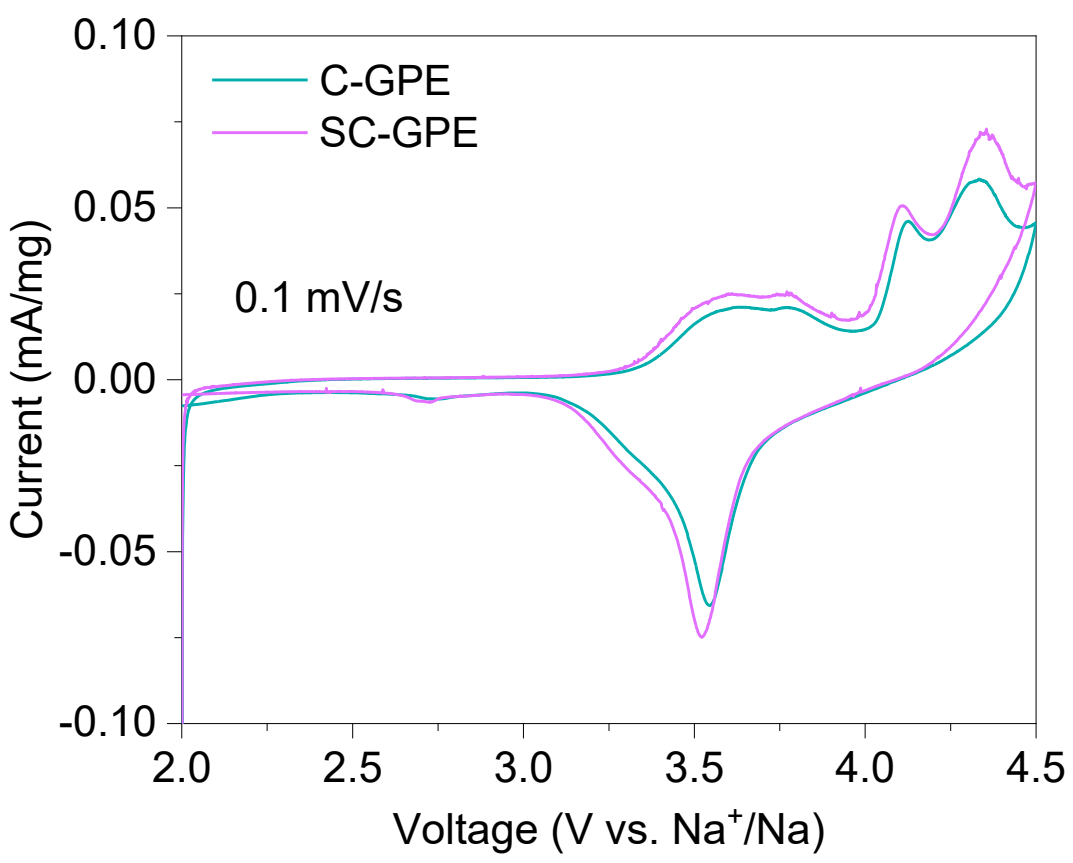

Supplementary Fig. 20 Cyclic voltammetry curves of the Na||NFS cells using C-GPE and SC-

GPE between 2.0-4.5 V.

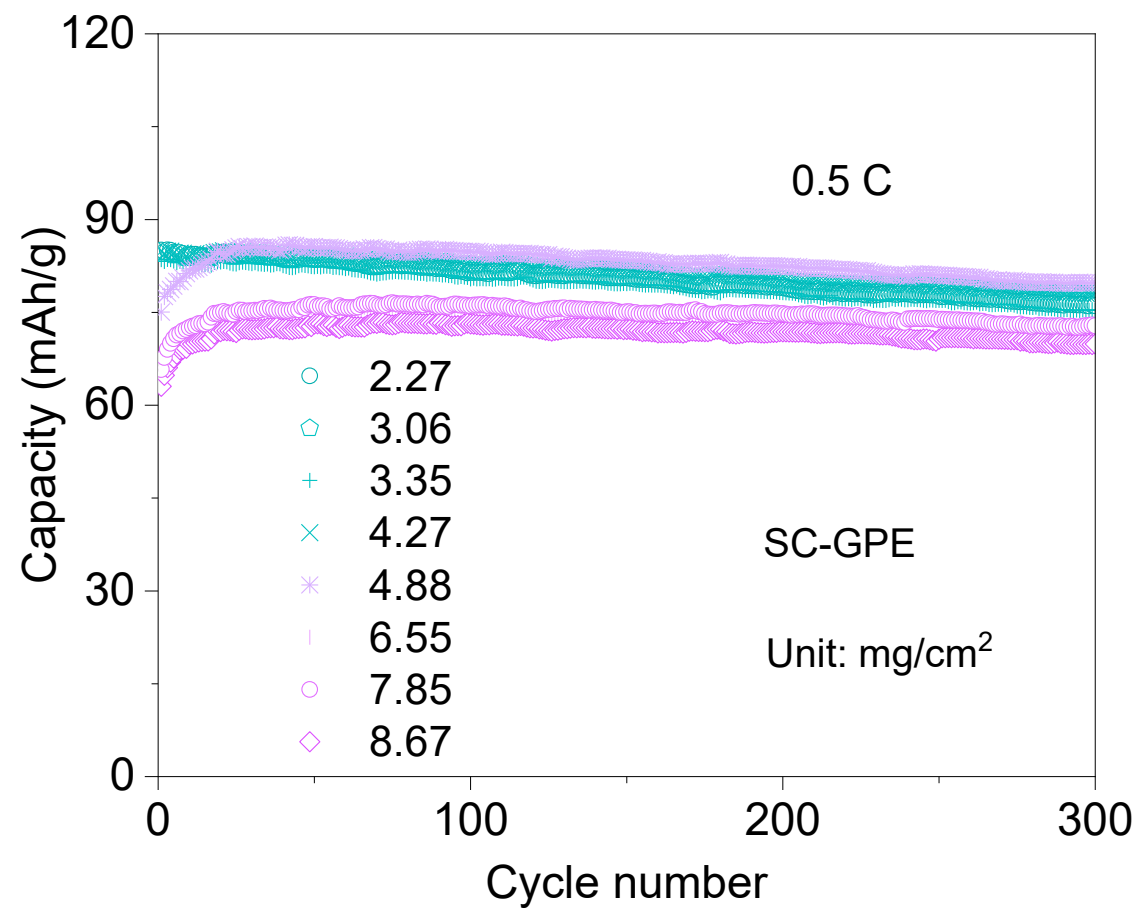

Supplementary Fig. 21 Cycling performance of the Na||NFS cells using SC-GPE at 0.5 C (1 C=120 mA g^{-1}) and 25°C.

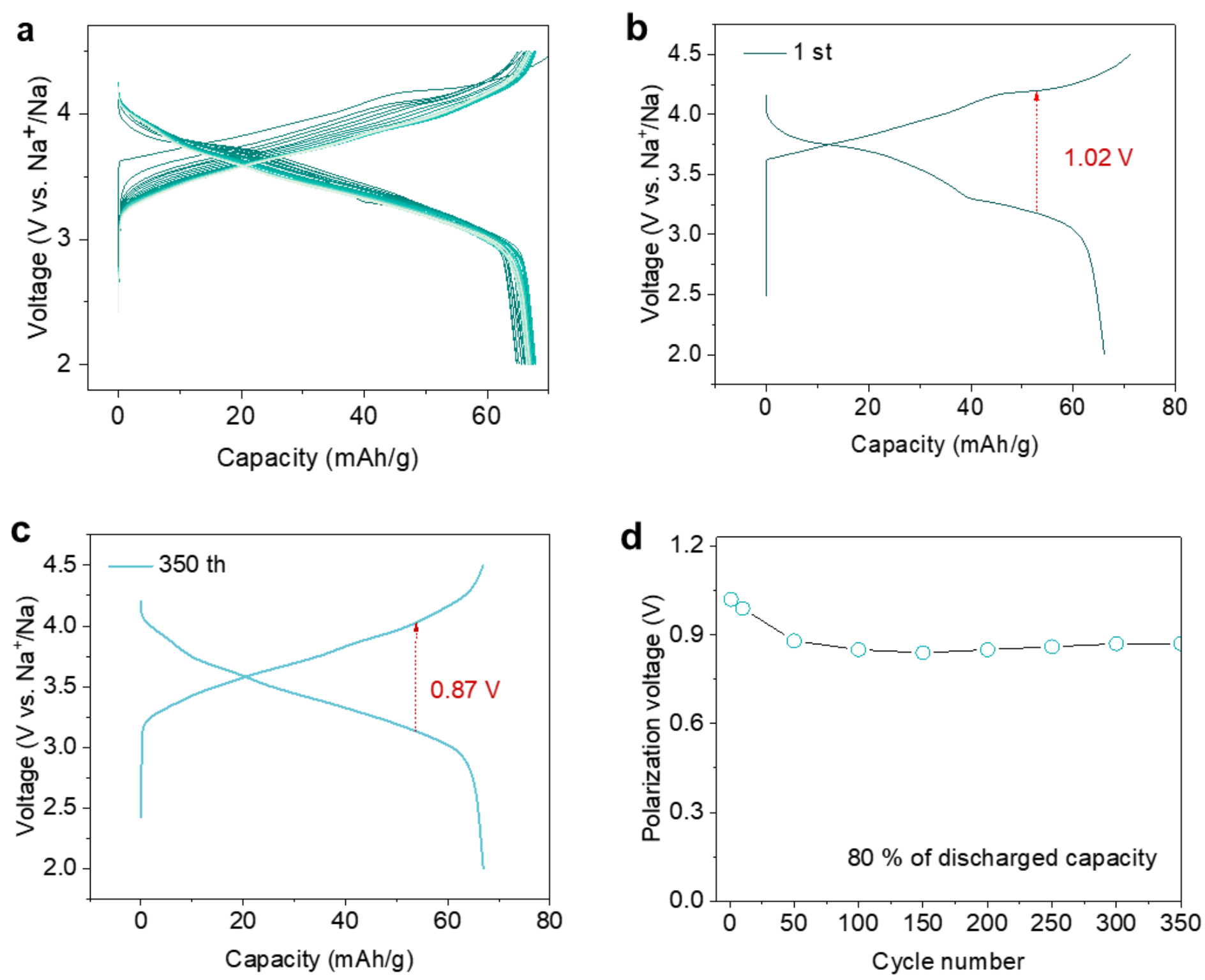

Supplementary Fig. 22 (a-c) Capacity-voltage plots of Na||NFS cell using SC-GPE and (d) their polarization voltage at 80% of discharged capacity during 350 cycles. The reduced polarization

voltage indicates that in the early stage of cycling, the NFS cathode/SC-GPE interface is mainly penetrated by a small amount of EC/PC/FEC solvent molecules. In the later stage of cycling, the PVDF-HFP chain dynamically penetrates between the NFS particles, achieving smooth transport of Na^+ and stable operation of the battery.

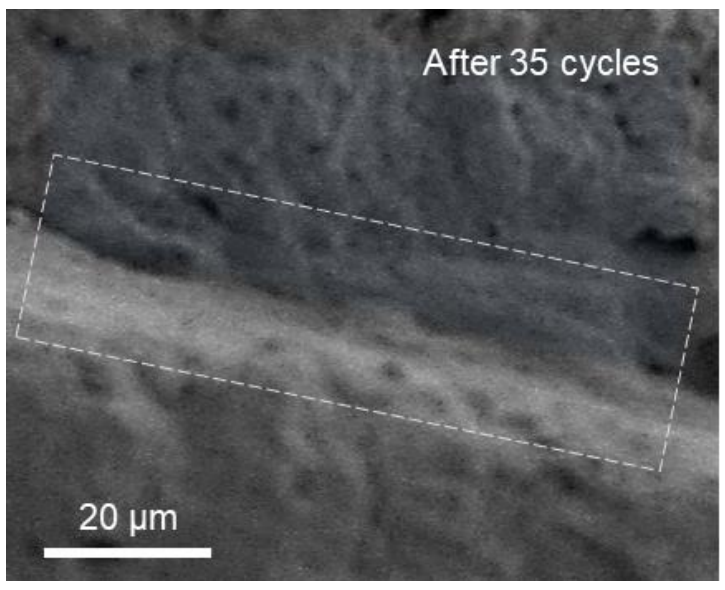

Supplementary Fig. 23 Cross sectional SEM image of SC-GPE after 35 cycles in Na||NFS cell.

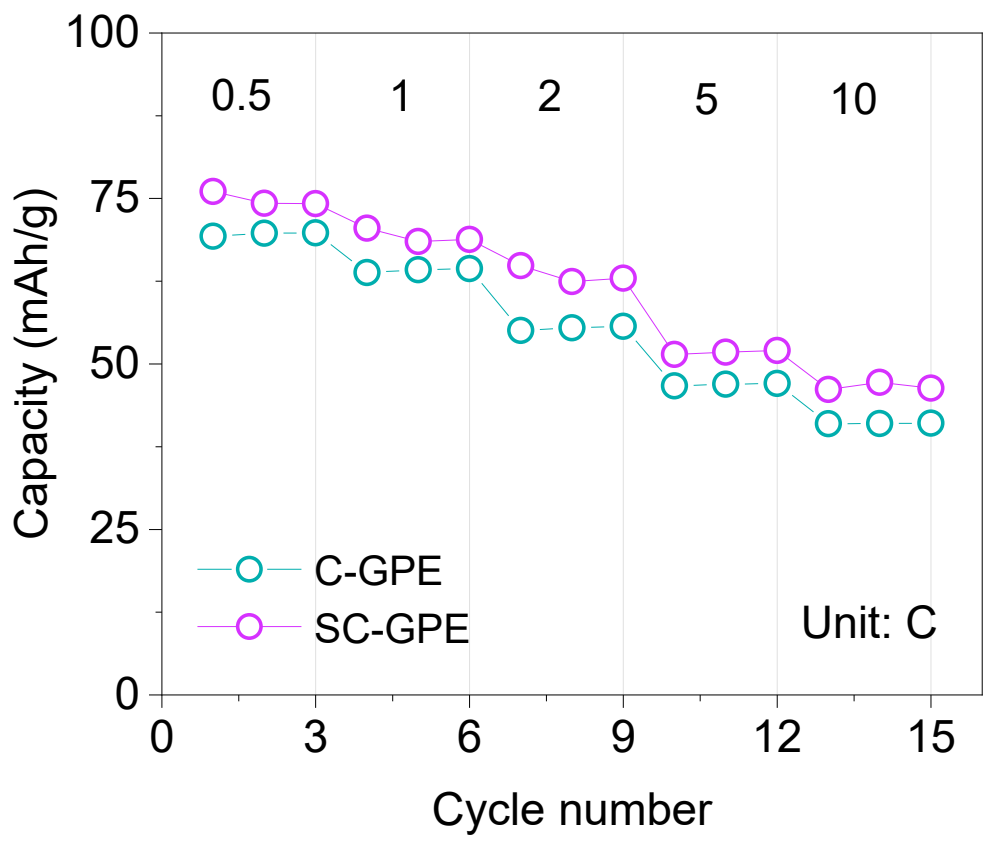

Supplementary Fig. 24 The capacity of the Na||NFS cells using C-GPE and SC-GPE at 0.5, 1, 2, 5, and 10 C for 3 cycles (1 C=120 mA g^{-1}).

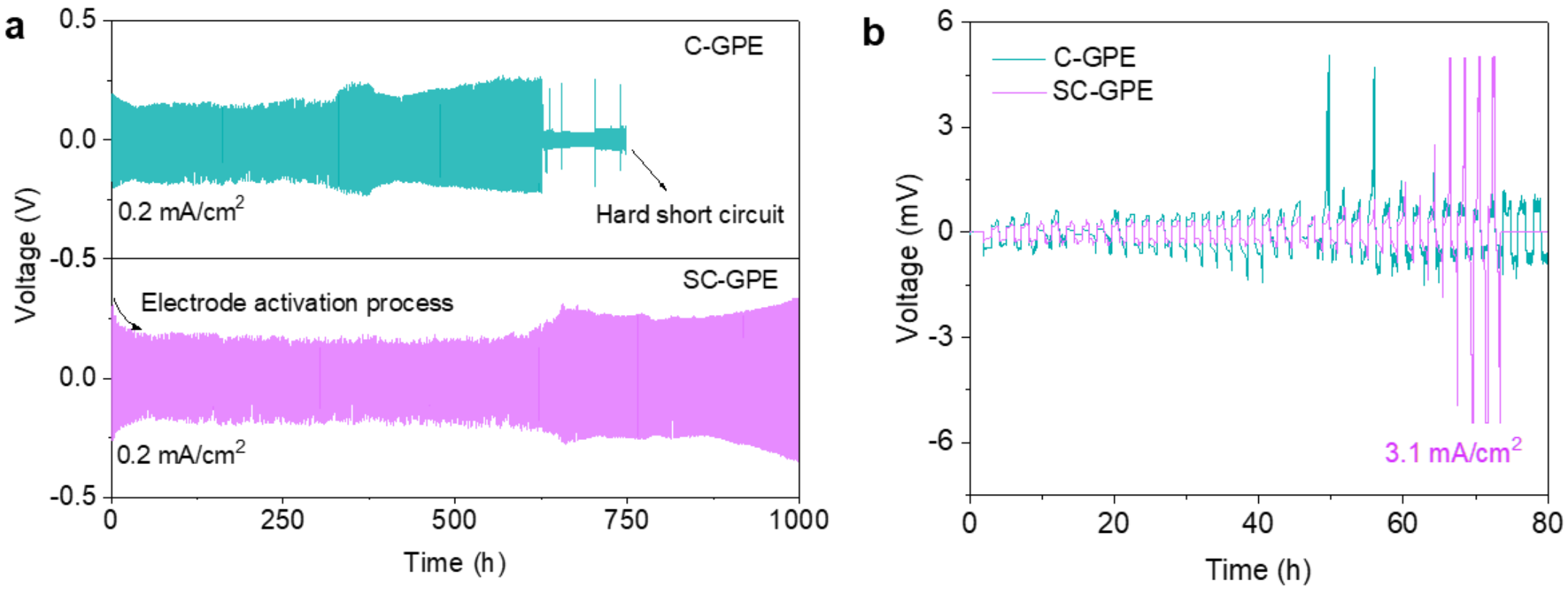

Supplementary Fig. 25 (a) Galvanostatic cycling profiles of the Na||Na cells using C-GPE and SC-GPE at 0.2 mA cm^{-2}. (b) Critical current density test for C-GPE and SC-GPE.

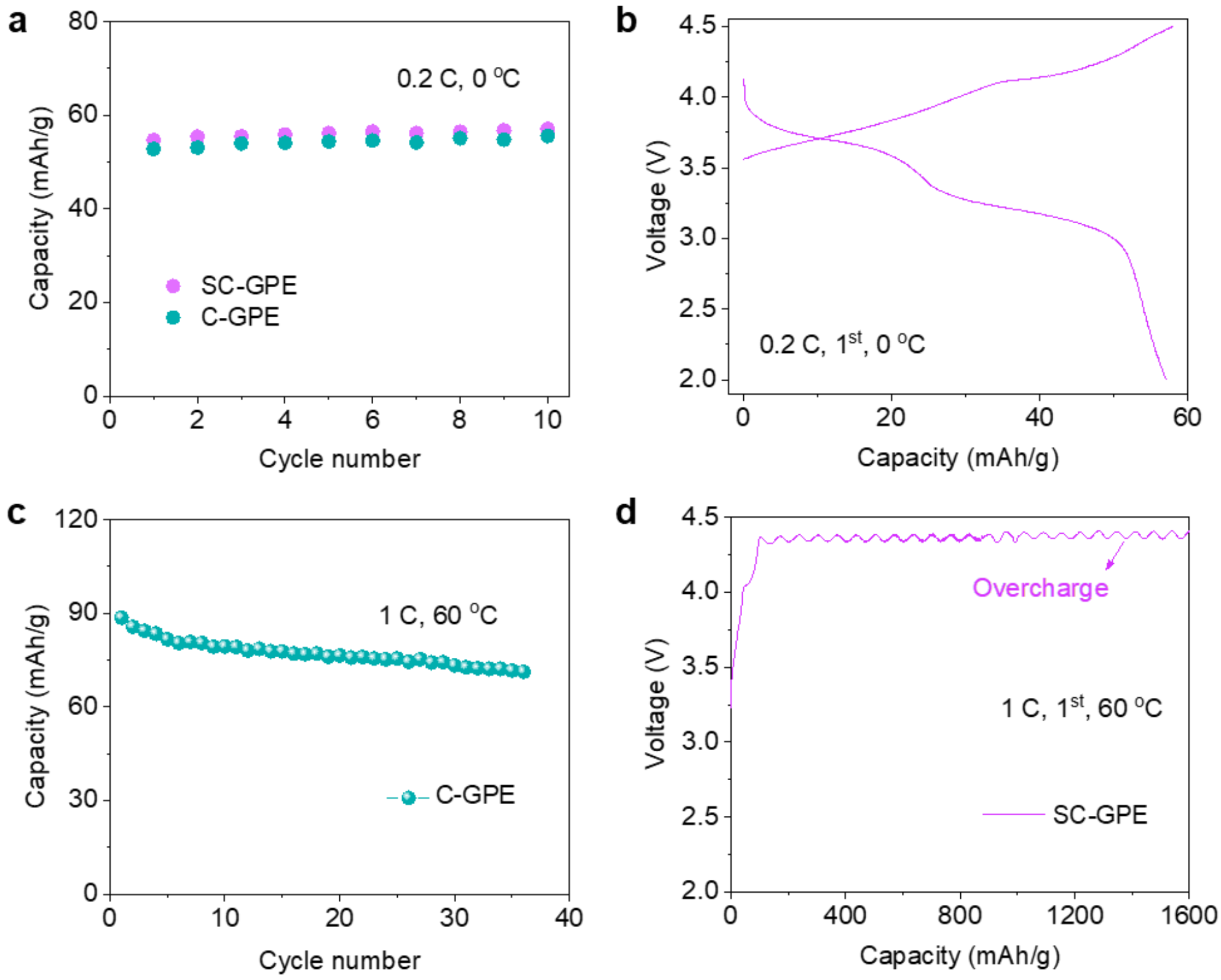

Supplementary Fig. 26 (a) Cycle performance of Na||NFS at 0.2 C, 0 °C and (b) the initial capacity-voltage curve of SC-GPE assembled Na||NFS cell, (c) Cycle performance of Na||NFS at 1 C, 60 °C and (d) the initial charge curve of SC-GPE assembled Na||NFS cell. At 0.2 C, 0 °C, and 2.0–4.5 V, similar bulk ion kinetics led to comparable discharge capacities for C-GPE and SC-GPE cells, delivering 52.8 and 54.7 mAh/g, respectively (Supplementary Fig. 26a, b). At 1 C and 60 °C, the C-GPE cell operated stably, with an initial capacity of 88.6 mAh/g and a retained capacity of 71.3 mAh/g after 36 cycles (Supplementary Fig. 26c). In contrast, the SC-GPE cell failed to operate properly, exhibiting

severe overcharging in the first cycle (Supplementary Fig. 26d). According to the temperature-dependent equation P=S×D, an increase in temperature raises P, facilitating DGM transport to the high-voltage NFS surface and causing sustained oxidative decomposition that results in severe overcharging.

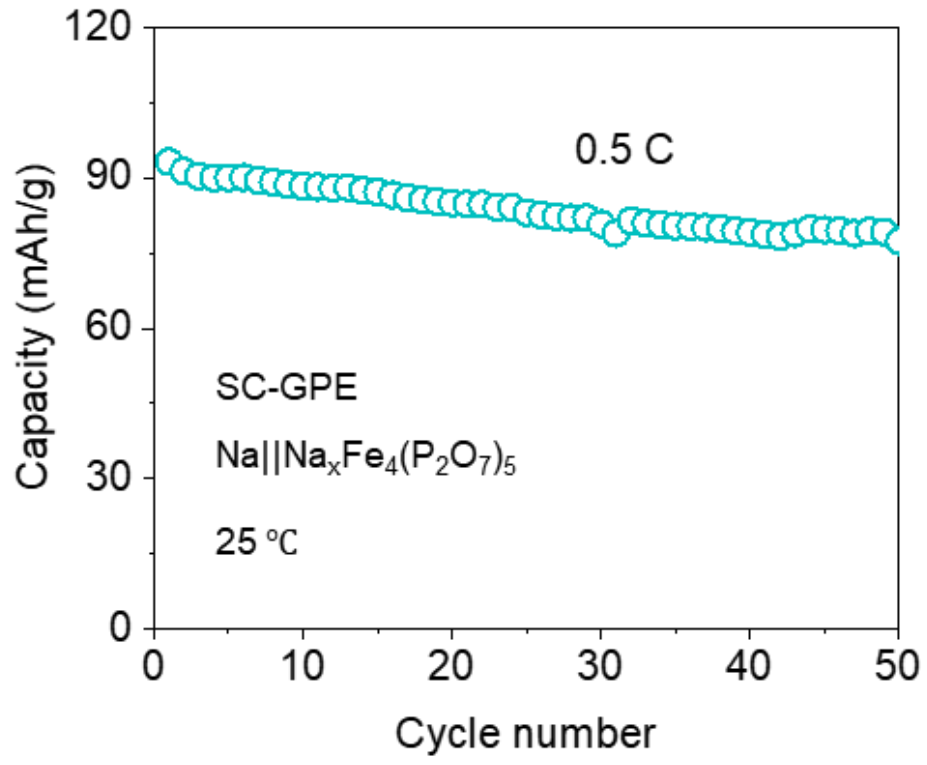

Supplementary Fig. 27 Cycling performance of the Na||$Na_xFe_4(P_2O_7)_5$ cell using SC-GPE at 0.5 C (1 C=120 mA g^{-1}) and 25 °C.

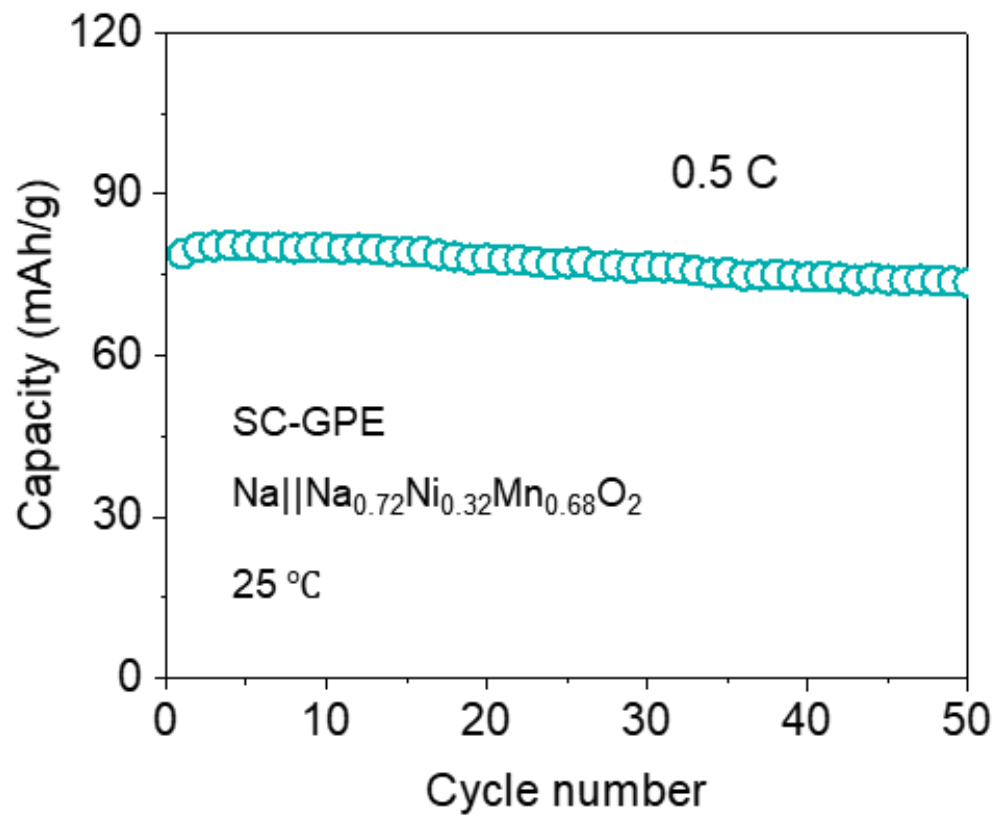

Supplementary Fig. 28 Cycling performance of the Na||$Na_{0.72}Ni_{0.32}Mn_{0.68}O_2$ cell using SC-GPE at 0.5 C (1 C=120 mA g^{-1}) and 25 °C.

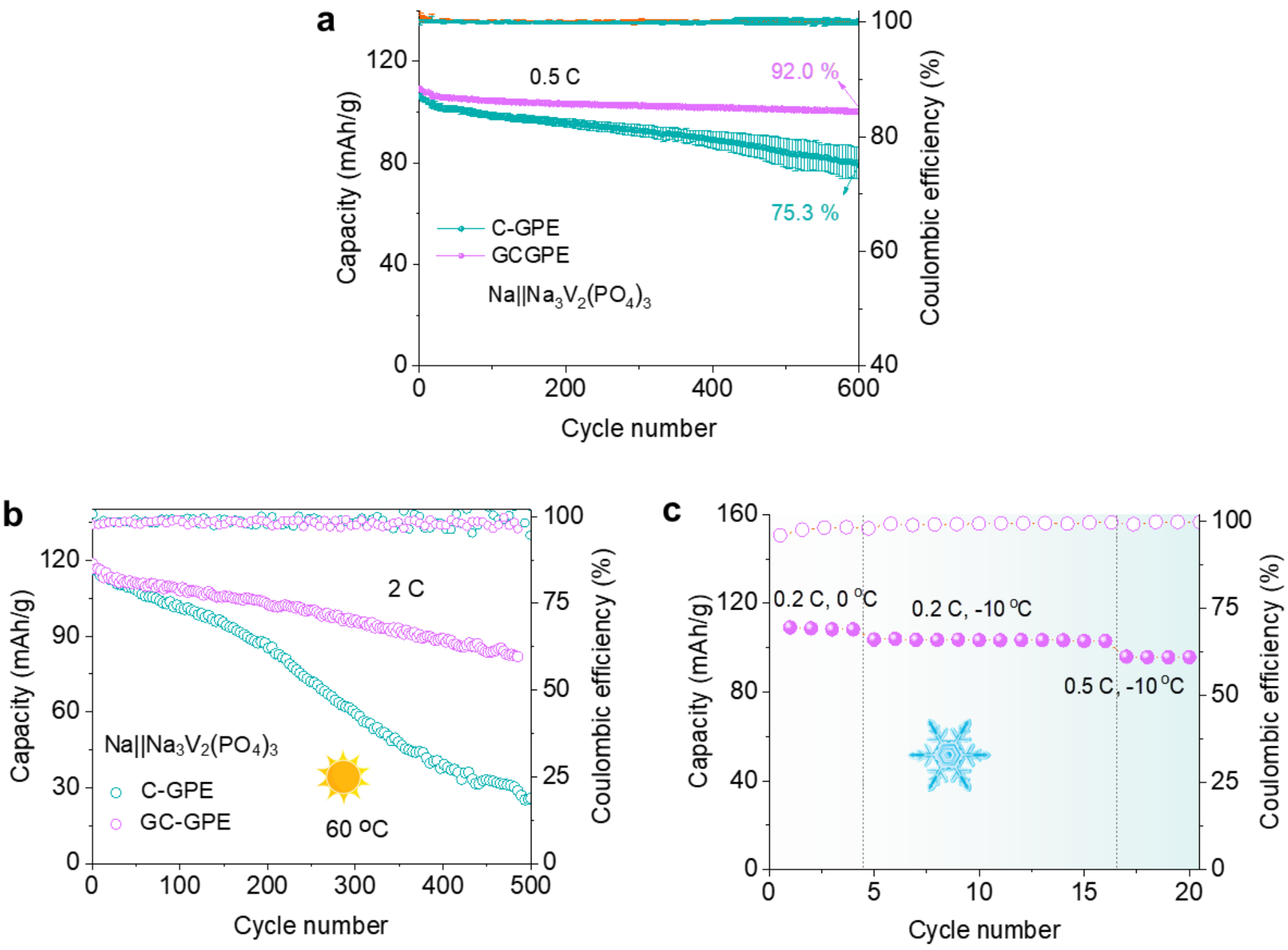

Supplementary Fig. 29 The discharge capacity of the Na||NVP cells using C-GPE and SC-GPE at (a) ambient temperature (25 °C) and 0.5 C, (b) low-temperature (-10-0 °C), 0.2 C and 0.5 C, and (c) high-temperature (60 °C), 2 C.

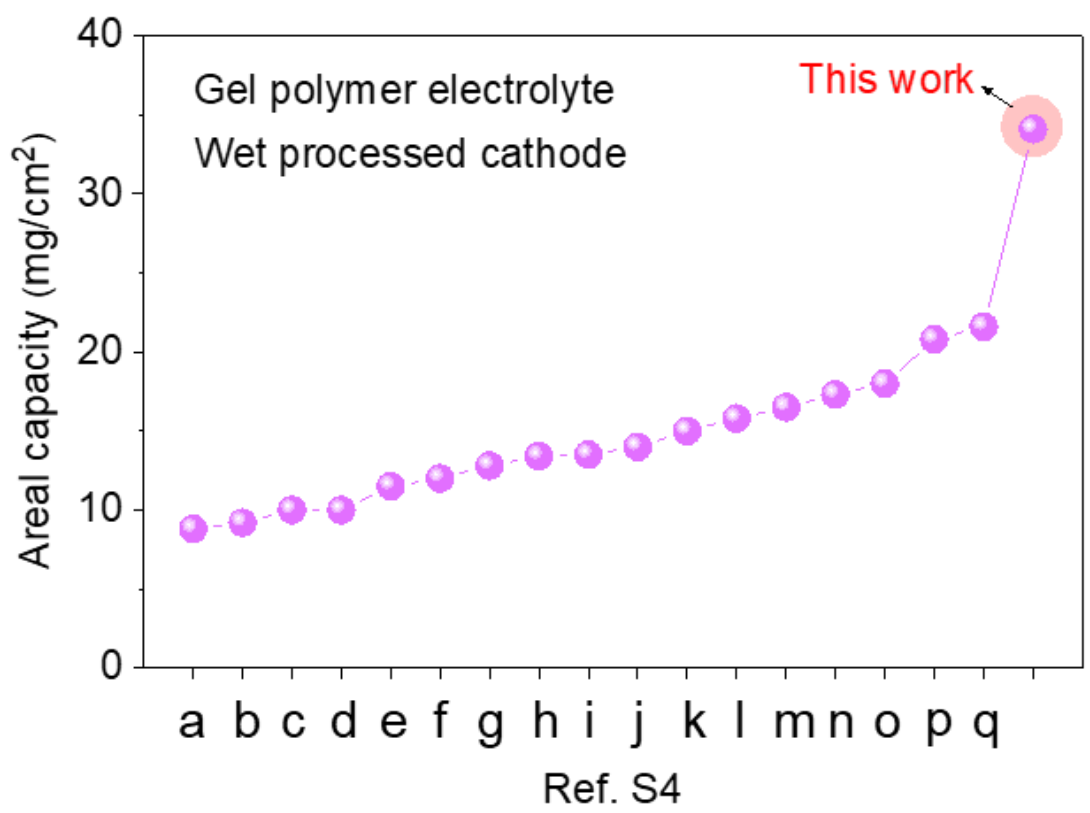

Supplementary Fig. 30 Comparison of cathode areal loadings with other reported works.

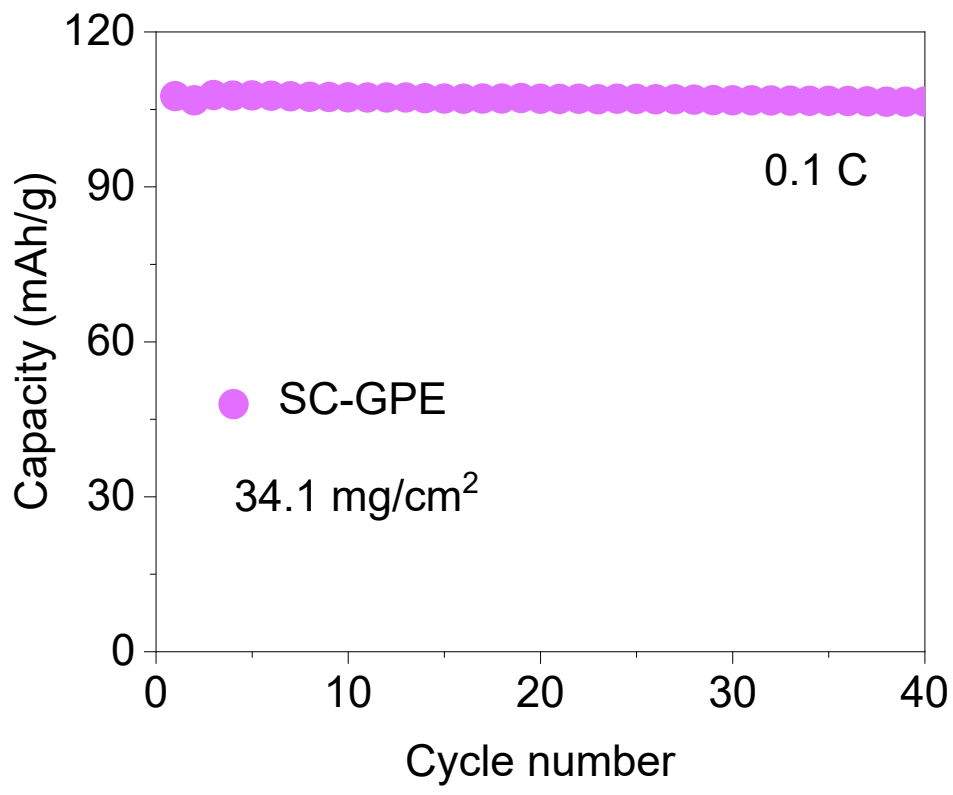

Supplementary Fig. 31 The cycling performance of Na||NVP coin cell using SC-GPE under 0.1 C.

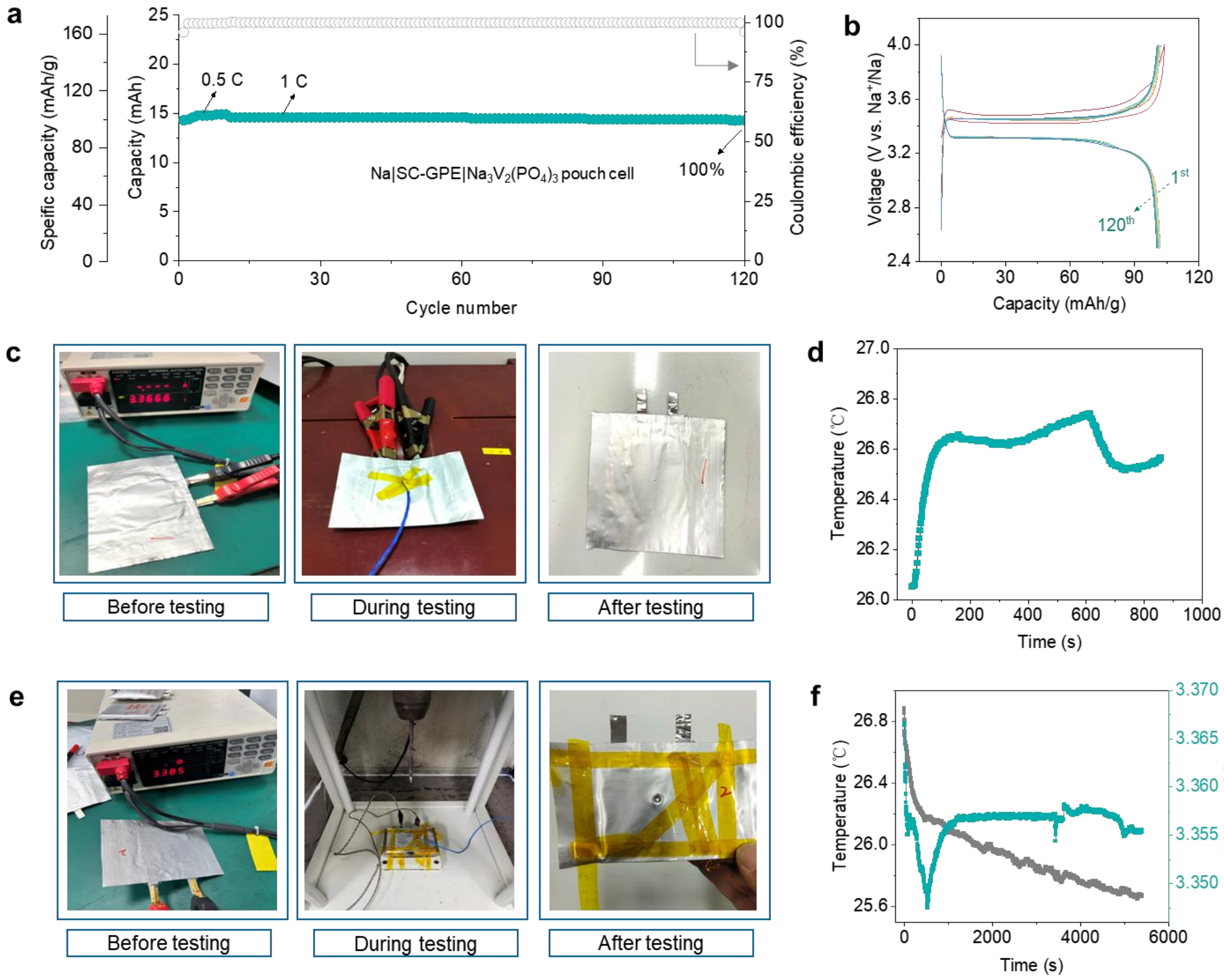

Supplementary Fig. 32 (a) The cycling performance of Na||NVP pouch cell using SC-GPE and (b) their capacity-voltage curves. (c) Photos of Na||NVP pouch cell under external short-circuit conditions and (d) temperature changes during the testing process. (e) Battery photos and (f) voltage and temperature changes during needle puncture testing of Na||NVP pouch cell.

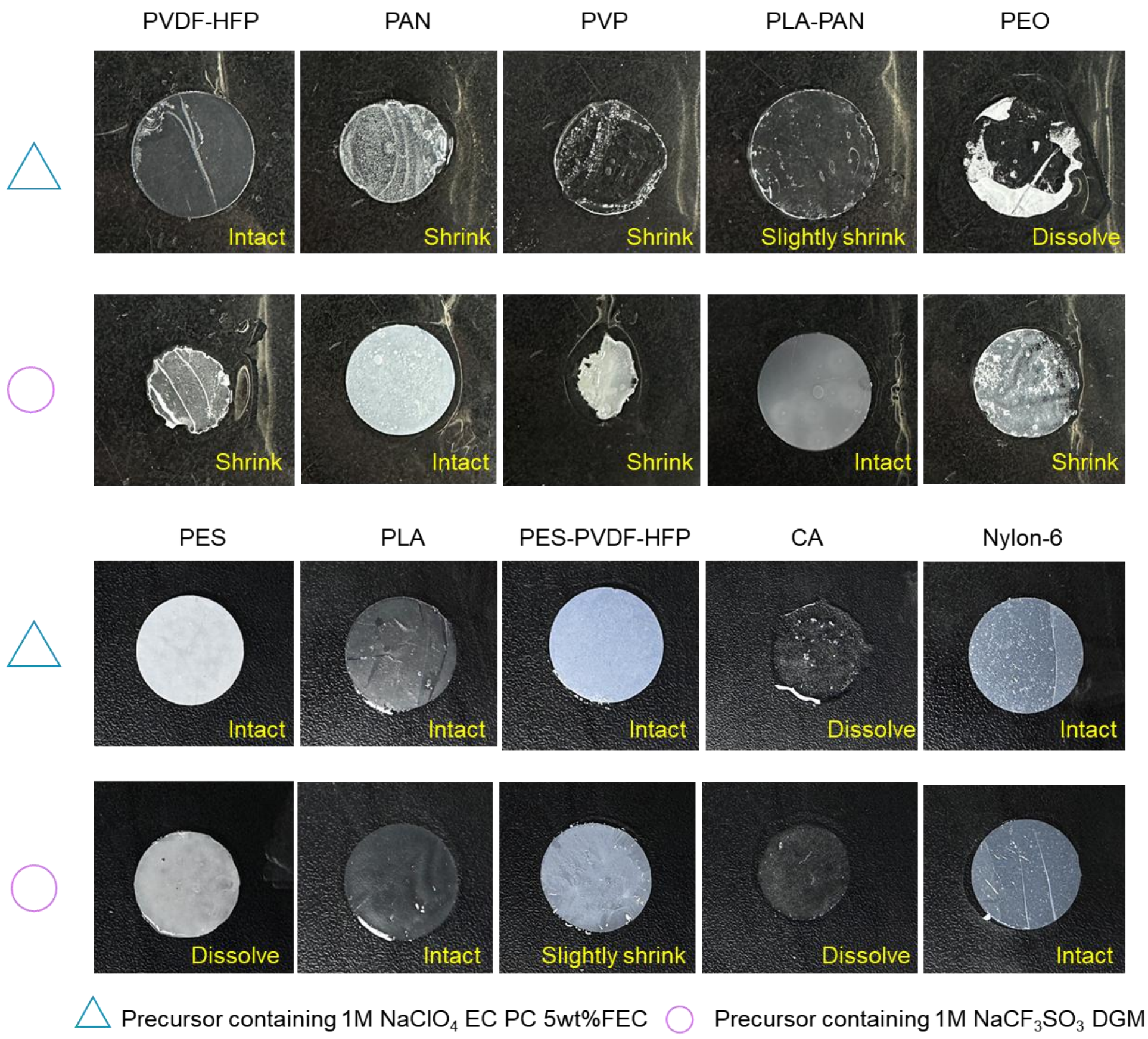

Supplementary Fig. 33 Stability tests between the polymer matrix and precursors.

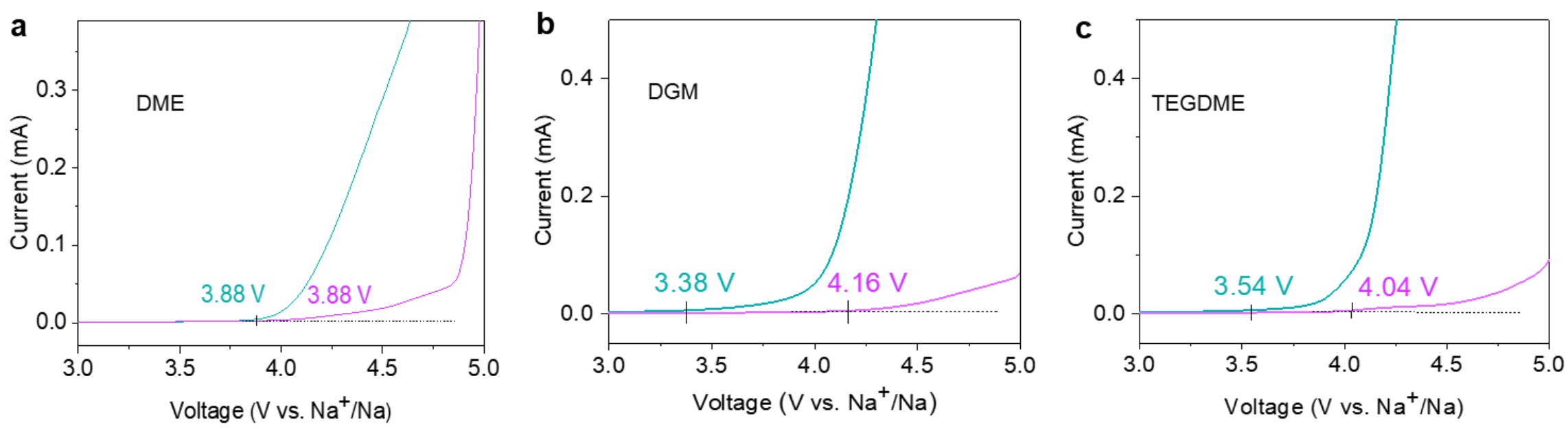

Supplementary Fig. 34 The LSV curves of Na||steel sheet cells using SC-GPE with different ether molecules in A-GPE.

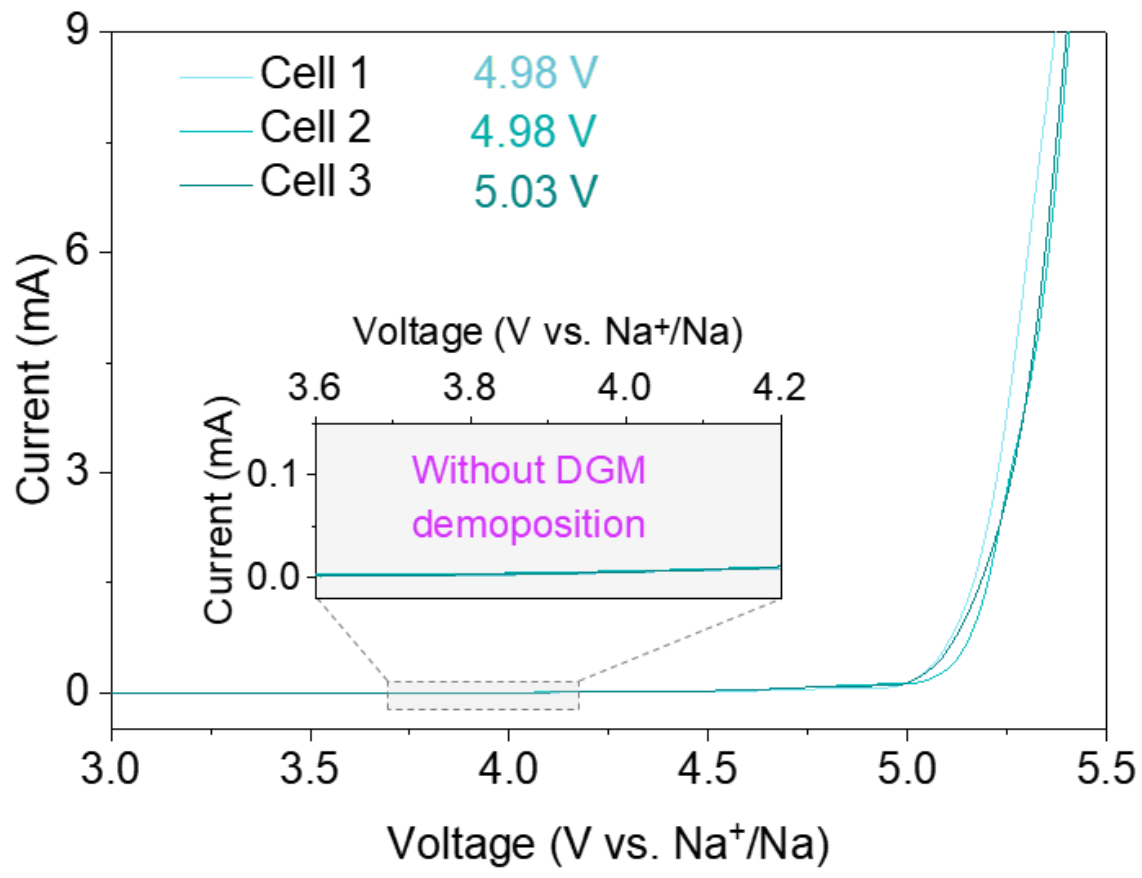

Supplementary Fig. 35 Three repeated LSV curves of Na||steel sheet cells using SC-GPE, and the inset is the enlarged region between 3.7 V and 4.2 V.

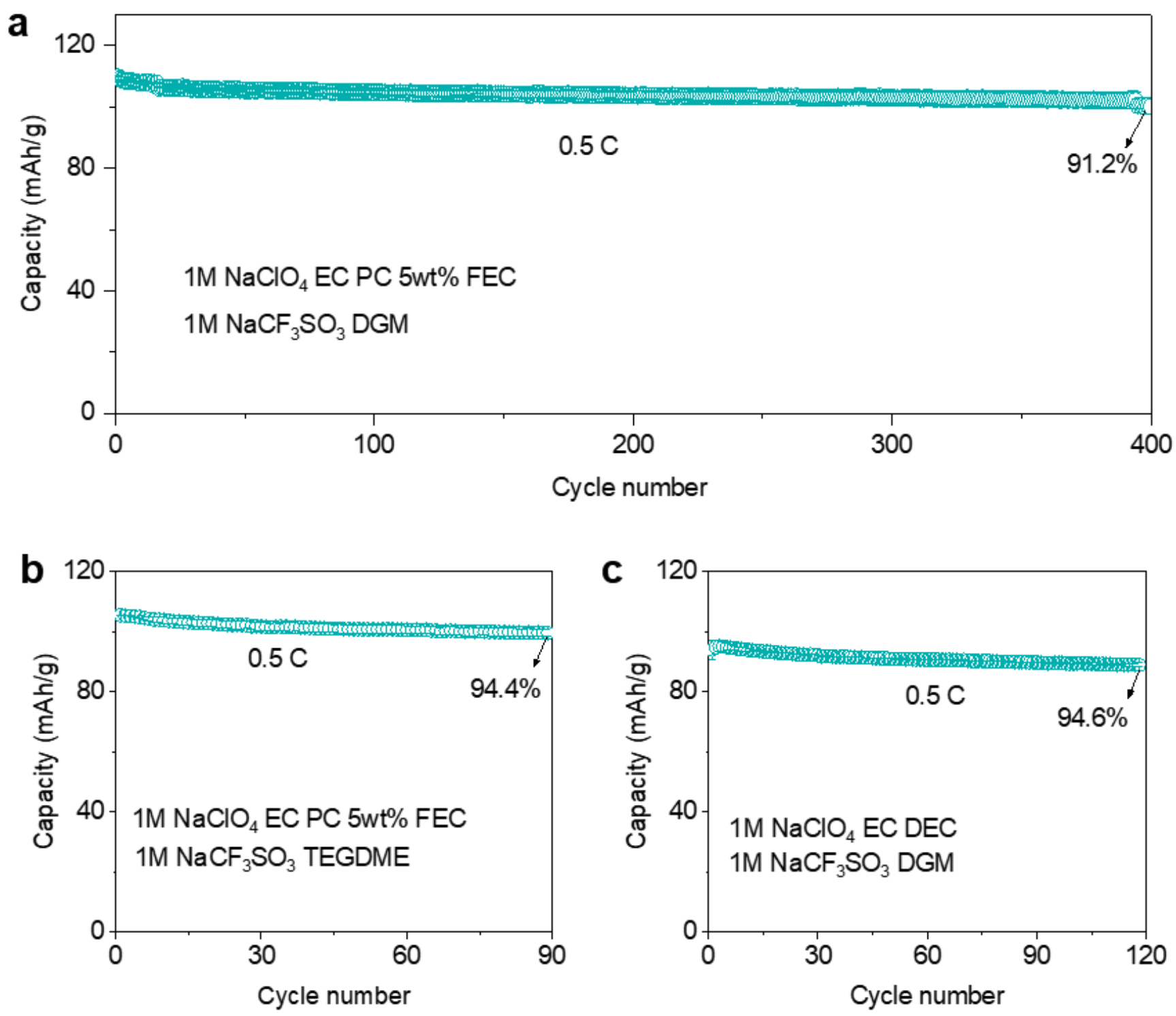

Supplementary Fig. 36 Cycling performance of the Na||NVP cells using SC-GPE with different electrolytes at 0.5 C, (1 C=117 mA g^{-1}).

Supplementary Tab.1 Summary of the self-extinguishing time between liquid electrolytes.

	Systems	Self-extinguishing time (SET, s g^{-1})	References
1	Liquid	137.6	S2a
	GPE	0	
2	Liquid	120	S2b
	FRGE	10	
3	Liquid	101.5	S2c
	LPE	10	
4	Liquid	62.5	S2d
	PFGPE	0	
5	Liquid	61.2	S2e
	LHCE-GPE	0	
6	Liquid	6	S2f
	FRGE	0.1	
7	GPE	0	S2g
8	TFGPE	0	S2h
9	PTALPT	0	S2i

Supplementary Tab.2 Comparison of the mass loadings and cycling stability with the previously reported polymer electrolytes.

	Mass loading (mg cm^{-2})	Capacity decay per cycle (mAh/g)	References
1	4.00	0.1210	S3a
2	2.00	0.0825	S3b
3	2.00	0.0510	S3c
4	1.00	0.0218	S3d
5	1.20	0.0166	S3e
6	2.50	0.0028	S3f
7	2.50	0.0012	S3g
8	5.82	~0	S3h
9	13.10	0.0200	S3i
10	14.00	0.0134	S3j
**11**	**16.40**	**~0**	**This work**

Supplementary Tab.3 Summary of the parameters of 191.3 Wh/kg Na||NFS pouch cell.

Parameter	Value for the Na||NFS pouch cell

Specific capacity	65.1 mAh/g
NFS mass loading (80.0 wt%)	18.7 mg/cm^2
Area capacity	2.2 mAh/cm^2
Active area	3.4 cm×3.0 cm
N/P ratio	3.62
Weight of Na anode	38.8 mg
Weight of NVP cathode	191.2 mg
Capacity	12.5 mAh
Energy	0.044 Wh
Energy density (Based on the mass of active materials)	191.3 Wh/kg

Supplementary Tab.4 Comparison of cathode areal loadings based on gel polymer electrolyte with other reported works.

**Cathode**	**Areal loadings (mg/cm^2)**	**Preparation Method**	**References**
$LiNi_{0.6}Co_{0.2}Mn_{0.2}O_2$	8.8	Wet processed	S4a
$LiNi_{0.8}Co_{0.1}Mn_{0.1}O_2$	9.2	Wet processed	S4b
$LiNi_{0.8}Co_{0.1}Mn_{0.1}O_2$ /$LiFePO_4$	10.0/10.0	Wet processed	S4c
$LiFePO_4$	10.0	Wet processed	S4d
$LiNi_{0.8}Co_{0.1}Mn_{0.1}O_2$	11.3	Wet processed	S4e
$LiFePO_4$	11.5	Wet processed	S4f
$LiFePO_4$	12.0	Wet processed	S4g
$LiFePO_4$	12.8	Wet processed	S4h
$LiFe_{0.4}Mn_{0.6}PO_4$	13.4	-	S4i
$Na_3V_2(PO_4)_3$	13.5	Wet processed	S4j
O3-$NaNi_{1/3}Fe_{1/3}Mn_{1/3}O_2$	14.0	Wet processed	S4k
$LiNi_{0.8}Co_{0.1}Mn_{0.1}O_2$	15.0	Wet processed	S4l
$LiNi_{0.6}Co_{0.2}Mn_{0.2}O_2$	15.8	Wet processed	S4m
$LiFePO_4$	16.5	Wet processed	S4n
$LiNi_{0.8}Co_{0.1}Mn_{0.1}O_2$	17.3	Wet processed	S4o
$LiNi_{0.8}Co_{0.1}Mn_{0.1}O_2$	18.0	Wet processed	S4p
$LiNi_{0.6}Co_{0.2}Mn_{0.2}O_2$	20.8	Wet processed	S4q
$LiNi_{0.6}Co_{0.2}Mn_{0.2}O_2$	21.6	Wet processed	S4r
**$Na_3V_2(PO_4)_3$**	**34.1**	**Wet processed**	**This work**

6. References

(S1) Wen, L.; Zhang, J.; Zhang, J.; Zhao, L.; Wang, X.; Wang, S.; Ma, S.; Li, W.; Luo, J.; Ge, J.; Chen, W. *eScience,* **2024**, 10.1016/j.esci.2024.100313

(S2) (a) Cheng, X.; Lu, C.; Gong, X.; Li, C.; Wang, J.; Qu, J.; Zhang, Y.; Song, T.; Zhang, Y.; Jiang, H.; Wang, C.; Long, Y.; Cao, Y.; Wang, Y.; Li, W.; Peng, H.; Wang, B. *Angew. Chem. Int. Ed*., **2025**, *64*, e202423419. (b) Xue, L.; Pan, H.; Wang, Y.; Wang, Z.; Wang, X.; Lin, Qian.; Zhang, K.; Bu, X.; Bai, M.; Hong, B. *Chem. Eng. J*., **2025,** *504*, 158828. (c) Zhu, G.; Zhang, Q.; Liu, Q.; Bai, Q.; Quan, Y.; Gao, Y.; Wu, G.; Wang, Y. *Nat. Commun*., **2023**, *14*, 4617. (d) Yang, B.; Pan, Y.; Li, T.; Hu, A.; Li, K.; Li, B.; Yang, L.; Long, J.; *Energy Storage Mater*., **2024**, *65*, 103124. (e) Wang, L.; Qu, Y.; Su, C.; Li, R.; Liu, D.; Jiang, W.; Jin, X.; Pei, M.; Jian, X.; Hu, F.; *Adv. Energy Mater*., **2025**, *15*, 2501185. (f) Xue, L.; Pan, H.; Wang, Y.; Wang, Z.; Wang, X.; Lin, Q.; Zhang, K.; Bu, X.; Bai, M.; Hong, B. *Chem. Eng. J*., **2025**, *504*, 158828. (g) Li, C.; Jing, Y.; Zhu, J.; Lv, Q.; Huo, M.; Zhang, H.; Wang, L.; Liu, Y.; Liu, S.; Yin, M.; Wang, X.; Wang, D.; Liu, H.; Dou, S.; Li, H.; Wang, B. *Adv. Funct. Mater.,* **2025**, https://doi.org/10.1002/adfm.202522002. (h) Zhang, Y.; Li, Z.; Zhang, S.; Li, J.; Lei, S.; Dong, P.; Zeng, W. Wang, J.; Chen, X.; Li, D.; Mu, S. *Nano Lett*., **2025**, *25*, 4930. (i) Yang, H.; Chen, Y.; Tian, W.; Yuan, S.; Liu, P.; Wang, Q.; Jin, T.; Jiao, L. *Angew. Chem. Int. Ed*., **2025**, *64*, e202506349.

(S3) (a) Dong, X.; Zhang, Y.; You, Z.; Chen, Y.; Wu, X.; Wen, Z. *Adv. Funct. Mater.,* **2024**, 34, 2405931. (b) Zhao, S.; Shen, Y.; Che, H.; Jabeen, M.; Lu, C.; Liao, X.; Ma, Z. *Adv. Funct. Mater.,* **2024**, 10.1002/adfm.202422162. (c) Yang, Z.; Jiang, H.; Li, X.; Liang, X.; Wei, J.; Xie, Z.; Tang, B.; Zhang, Y.; Zhou, Z. *Adv. Funct, Mater.,* **2024**, *34*, 407713. (d) Su, C.; Qu, Y.; Hu, N.; Wang, L.; Song, Z.; Pei, M.; Mao, R.; Jin, X.; Liu, D.; Jian, X.; Hu, F. *Angew. Chem. Int. Ed.,* **2025**, 10.1002/anie.202418959. (e) Pan, J.; Zhang, Y.; Sun, F.; Osenberg, M.; Hilger, A.; Manke, I.; Cao, R.; Dou, S.; Fan, H. *Angew. Chem. Int. Ed.,* **2023**, *62*, e202219000. (f) Zhou, Y.; Xiao, Z.; Han, D.; Yang, L.; Zhang, J.; Tang, W.; Shu, C.; Peng, C.; Zhou, D. *Adv. Funct. Mater.,* **2022**, *32*, 2111314. (g) Guo, J.; Feng, F.; Zhao, S.; Wang, R.; Yang, M.; Shi, Z.; Ren, Y.; Ma, Z.; Chen, S.; Liu, T. *Small,* **2023**, *19*, 2206740. (h) Fan, Y.; Feng, Y.; Li, G.; Bo, Y.; Wang, C.; Wang, D.; Qian, Y.; Ma, R.; Hu, Z.; Zhang, K.; Chen, J. *Adv. Mater.,* **2024**, *36*, 2413303. (i) Yang, M.; Feng, F.; Ren, Y.; Chen, S.; Chen, F.; Chu, D.; Guo, J.; Shi, Z.; Cai, T.; Zhang, W.; Ma, Z.; Chen, S.; Liu, T. *Adv. Funct. Mater.,* **2023**, *33*, 2305383. (j) Zhang, J.; Wang, Y.; Xia, Q.; Li, X.; Liu, B.; Hu, T.; Tebyetekerwa, M.; Hu, S.; Knibbe, R.; Chou, S. *Angew. Chem. Int. Ed.,* **2024**, *63,* *e202318822.*

(S4) (a) Ye, S.; Zhang, Y.; Huang, Y.; Li, Y.; Li, Z.; Ou, C.; Lin, M.; Tian, F.; Lei, D.; Wang, C. *Angew. Chem. Int. Ed.,* **2025**, *64*, e202506662. (b) Zhang, S.; Li, Z.; Zhang, Y.; Wang, X.; Dong, P.; Lei, S.; Zeng, W.; Wang, J.; Liao, X.; Chen, X.; Li, D.; Mu, S. *Energy Environ. Sci.,* **2025**, *18*, 3807. (c) Li, M.; Rakov, D. A.; Fan, Y.; Wang, C.; Wang, C.; Yuwono, J. A.; Xia, S.; Mao, J.; Guo, Z. *Angew. Chem. Int. Ed.,* **2025**, *64*, e202513450. (d) Zhai, Y.; Zhang, Y.; Wang, N.; Li, M.; Shen, M.; Xu, P.; Sun, M.; Dong, L.; Ma, T.; Ming, J.; Cong, L.; Xie, H.; Liu, Y. *Energy Environ. Sci.*, 2025, *18*, 8352. (e) Zhou, X.; Chen, Y.; Zhen, F.; Wu, Y.; Li, W.; Yin, X.; Liu, L.; Ding, S.; Yu, W. *ACS Nano,* **2025**, *19*, 14073. (f) Gou, J.; Zhang, Z.; Wang, S.; Huang, J.; Cui, K.; Wang, H. *Adv. Mater.,* **2024**, *36*, 2309677. (g) Liu, X.; Wang, D.; Zhang, Z.; Li, G.; Wang, J.; Yang, G.; Lin, H.; Lin, J.; Ou, X.; Zheng, W. *Small,* **2024**, *20*, 2404879. (h) Feng, Y.; Fan, Y.; Zhao, L.; Yu, J.; Liao, Y.; Zhang, T.; Zhang, R.; Zhu, H.; Sun, X.; Hu, Z.; Zhang, K.; Chen, J. *Angew. Chem. Int. Ed.,* **2025**, *64*, e202417105. (i) Nie, L.; Gao, R.; Zhang, M.; Zhu, Y.; Wu, X.; Lao, Z.; Zhou, G. *Adv. Energy Mater.,*

2023, *14*, 2302476. (j) Guo, X.; Xie, Z.; Wang, R.; Luo, J.; Chen, J.; Guo, S.; Tang, G.; Shi, Y.; Chen, W. *Angew. Chem. Int. Ed.,* **2024**, *63*, e202402245. (k) Du, L.; Xu, G.; Sun, C.; Zhang, Y.; Zhang, H.; Dong, T.; Huang, L.; Ma, J.; Sun, F.; Li, C.; Zhuang, X.; Zhang, S.; Li, J.; Xie, B.; Wang, J.; Zhao, J.; Ju, J.; Hu, Z.; Chang, F.; Kuo, C.; Chen, C.; Hilger, A.; Manke, I.; Dong, S.; Cui, G. *Nat. Commun.,* **2025**, *16*, 2979. (l) Shi, Y.; Miao, X.; Zhao, G.; Xiao, S.; Guo, Z. *Adv. Funct. Mater.,* 10.1002/adfm.202524608. (m) Chen, J.; He, C.; Peng, X.; Li, J.; Xu, X.; Zhou, Y.; Shen, J.; Sun, J.; Li, Y.; Zhao, T. *Nat. Commun.,* **2025**, *16*, 8494. (n) Wang, P.; Xie, H.; Liu, Y.; Bai, Z.; Li, N.; Hong, S.; Zhou, C.; Shan, L.; Zhao, Z.; Shi, X. *Adv. Energy Mater.,* 10.1002/aenm.202506710. (o) Feng, J.; Wang, J.; Gu, Q.; Li, P.; Xu, H.; Deng, Y.; Gao, P. *Adv. Funct. Mater.,* **2025**, *35*, 2412287. (p) Huang, L.; Wu, Y.; Li, M.; Zeng, B.; Liang, J.; Song, X.; Su, K.; Luo, P.; Song, H.; Cui, Z. *Mater. Today,* **2025**, *91*, 176. (q) Xu, N.; Zhao, Y.; Ni, M.; Zhu, J.; Song, X.; Bi, X.; Zhang, J.; Zhang, H.; Ma, Y.; Li, C.; Chen, Y. *Angew. Chem. Int. Ed.*, **2024**, *63*, e202404400. (r) Li, J.; Chen, J.; Xu, X.; Wang, Z.; Guo, Z.; Lin, P.; Sun, J.; Huang, B.; Zhao, T. *Sci. Adv.,* **2025**, *11*, eadz5203.
